# Rice cycles between drought and well-watered-adapted phenotypes by changing lateral root formation

**DOI:** 10.1093/aob/mcaf173

**Published:** 2025-08-12

**Authors:** Helena Bochmann, Marie Klein, Amelia Henry, Heike Faßbender, Marjorie De Ocampo, Josefine Kant, Michelle Watt

**Affiliations:** Institute of Bio- and Geosciences, IBG-2: Plant Sciences, Forschungszentrum Jülich GmbH, 52425 Jülich, Germany; Institute of Crop Science and Resource Conservation, Faculty of Agricultural, Nutritional and Engineering Sciences, University of Bonn, 53115 Bonn, Germany; Institute of Bio- and Geosciences, IBG-2: Plant Sciences, Forschungszentrum Jülich GmbH, 52425 Jülich, Germany; Rice Breeding Innovations Department, International Rice Research Institute, Pili Drive, Los Baños, Laguna 4031, Philippines; Institute of Bio- and Geosciences, IBG-2: Plant Sciences, Forschungszentrum Jülich GmbH, 52425 Jülich, Germany; Rice Breeding Innovations Department, International Rice Research Institute, Pili Drive, Los Baños, Laguna 4031, Philippines; Institute of Bio- and Geosciences, IBG-2: Plant Sciences, Forschungszentrum Jülich GmbH, 52425 Jülich, Germany; Institute of Bio- and Geosciences, IBG-2: Plant Sciences, Forschungszentrum Jülich GmbH, 52425 Jülich, Germany; School of Biosciences, University of Melbourne, Parkville, Victoria 3010, Australia

**Keywords:** *Oryza sativa*, drought, drought recovery, climate change, root architecture, lateral roots

## Abstract

**Background and Aims:**

Natural rainfed conditions present drought episodes interspersed with periods of moderate to high soil moisture levels. This study investigates the genetic variation in root-to-shoot growth in response to a wet–drought–wet cycle and aims to identify rice (*Oryza sativa*) lines differing in drought recovery, focusing on detailed root trait investigations.

**Methods:**

In total, 100 different rice accessions were screened under fluctuating moisture across three field seasons for GWAS (genome-wide association study) analysis. In a subset of 20 genotypes, crown root number and leaf length were recorded regularly to calculate a water recovery index (WRI). Two lines contrasting in WRI were grown in a glasshouse experiment to resolve detailed root phenotypes in simulated field drought and re-watering.

**Key Results:**

GWAS co-locations indicated drought recovery-associated loci that included candidate genes previously reported for several abiotic stressors. In the subset of 20 genotypes, crown root growth was impacted most by the transition from drought to re-watering. The calculated WRI distinguishes different responses to drought and re-watering. A glasshouse study reproduced the contrasting growth of two selected lines, with ‘ADT 12’ shoot and root growth being strongly impaired by drought, while ‘ARC 18202’ growth was not suppressed. Drought caused a significant decrease in S-type lateral root production in both lines, while a significant increase in L-type lateral root proportion was only found for ‘ADT 12’. These phenotypes were reversed 7 d after re-watering to values of the well-watered control plants.

**Conclusions:**

Overall, in-depth root phenotyping confirmed the drought-resistance and recovery ability of ‘ARC 18202’ in the field and highlighted the importance of S-type and L-type lateral root formation already under well-watered conditions prior to drought. ‘ARC 18202’ had a higher amount of thick lateral roots before drought and, therefore, less change in lateral root formation under drought and re-watering conditions.

## INTRODUCTION

Rice (*Oryza sativa* L.) provides the caloric majority for a large proportion of the global population. Large rice-growing areas are rainfed and thus prone to drought stress. Erratic rainfall patterns, climatic anomalies or periods of severe drought limit rice crop production in non-irrigated systems and motivate the need for more drought-stable rice varieties (Wassmann *et al*., 2009; Swapna and Shylaraj, 2017). New varieties are desired to contain adaptive traits such as seedling vigour, root plasticity and tolerance to moderate drought, including recovery (Heredia *et al*., 2022).

Drought response in rice can be described as escape, avoidance, tolerance and recovery, which was recently reviewed (Hassan *et al*., 2023). Plants that escape drought grow quicker to maturity, ending their life cycle before severe stress symptoms arise. Drought avoidance is defined by the maintenance of high water potential often accompanied by deep rooting, limited vegetative growth and higher water use efficiency. Drought tolerance describes rice plants that sustain morphological and physiological activities in water-limited conditions – stay green longer, greater accumulation of physiological protective substances (such as enzymes, osmo-protectants, reactive oxygen species scavengers), sustain photosynthesis, have higher hydraulic conductivity, and accumulate more soluble sugars and proline. The drought response also influences the ability of plants to recover. Recovery describes the ability of plants to restore growth after temporary drought events (Hassan *et al*., 2023). Recently, the importance of drought intensity and timing in the plant life cycle as well as adaptation has come into focus (Vadez *et al*., 2024). All of these criteria define how a genotype performs – its genetic and phenotypic response in one scenario may give more tolerance to drought, but may come with yield penalties in other drought types (Vadez *et al*., 2024).

The importance of drought recovery has been recognized for some time (Sanchez-Diaz and Kramer, 1971), yet compared to drought tolerance, relatively few studies have focused on drought recovery. These studies provide insights into shoot and root growth of different crops before and after re-watering. In rice, a series of pot experiments investigated the diversity of drought recovery responses by investigating above-ground growth traits, showing that a higher amount of green leaf biomass leads to faster leaf expansion after drought and better drought recovery (Wade *et al*., 2000). Drought caused roots to have a smaller diameter and increased specific root length (Azhiri-Sigari *et al*., 2000). After re-watering, as top-soil root growth increased, roots became thicker and the root-to-shoot ratio increased (Azhiri-Sigari *et al*., 2000). Root length density was positively correlated with water extraction in deeper soil layers under drought (Kamoshita *et al*., 2000). Moreover, critical root length densities were identified for rice for water extraction in pot studies under drought and re-watering (Siopongco *et al*., 2005). The ability to keep a comparable biomass growth during drought and well-watered conditions was identified as a goal for drought recovery (Kamoshita *et al*., 2004). Taken together, studies on rice drought recovery have indicated a key role of root traits conferring shoot growth recovery following drought. Thus, shoot and root growth measurements provide a basis for comparison of drought recovery. However, little is known about genetic variation in rice drought recovery strategies.

Rice has a complex root system containing specific root types. The seminal and crown roots of rice are the main axial roots producing lateral roots (LRs) (Yamauchi *et al*., 1987). The LRs are clearly distinguishable by diameter, with a larger diameter in L-type and smaller diameter in S-type LRs. In addition, L-type LRs grow longer and are capable of branching into higher order LRs while S-type LRs have a determined, shorter growth and stay unbranched (Yamauchi *et al*., 1987). Studies on the LRs of rice have indicated S-type LRs to have greater water conductance under drought stress (Watanabe *et al*., 2020 ) while L-type LRs were attributed to be more important for water and P uptake in drying soils (De Bauw *et al*., 2020).

In this study, rice drought recovery was tested in several field trials to perform a genome-wide association study (GWAS) based on shoot traits. Measuring below-ground root traits remains labour-intensive, as reviewed in Teramoto and Uga (2022). Therefore, we developed a water recovery index (WRI) as an indicator of a focus on stronger shoot or root growth during drought recovery in rice, using easily measured traits such as leaf elongation and number of crown roots. A high number of crown roots can be positively correlated with higher yield under drought (Siangliw *et al*., 2022). Finally, to include the complex response of the entire rice root system, a detailed glasshouse experiment was performed for in-depth root phenotyping of two WRI-contrasting lines.

In this study, we hypothesized that: (1) genetic association can be identified by GWAS screening for shoot variation in drought recovery; (2) different shoot-to-root ratios can be identified in drought and re-watering response strategies; and (3) variation in LR traits can distinguish these recovery strategies. By screening large numbers of diverse rice accessions for their drought recovery ability and characterizing their detailed root responses, we aimed to identify accessions that can become useful in breeding to improve the productivity and yield stability of rainfed rice.

## MATERIAL AND METHODS

### Field trials for GWAS

**Fig. 1.**
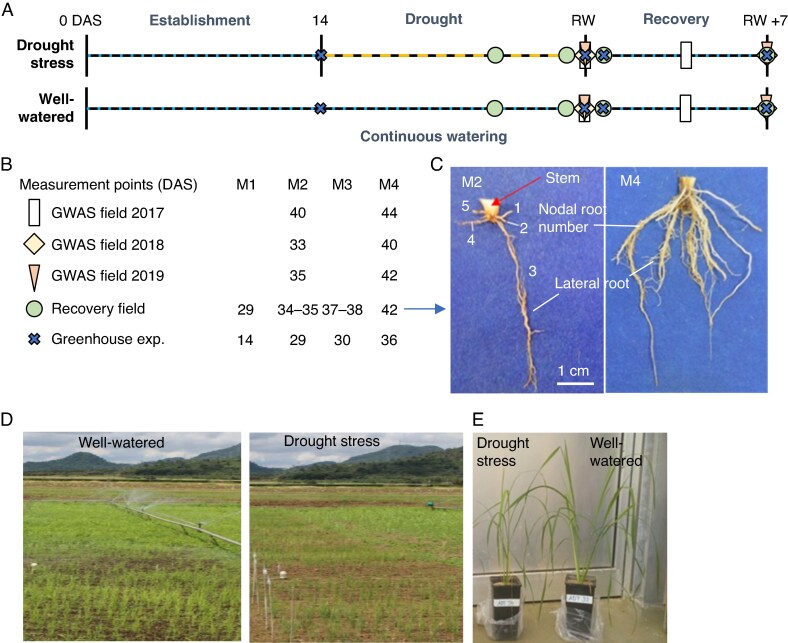
Experimental design of field and glasshouse experiments on rice drought recovery. (A) Timeline of experiments. Seedling establishment, recovery and continuous watering by sprinkler irrigation (field) or watering to weight (glasshouse), while drought was induced by withholding of water. The period of drought was variable in length due to climatic conditions with varying days-after-sowing (DAS) for re-watering (RW) as the end of drought, defined by shoot phenotyping (see Methods). (B) DAS for destructive measurement time points (M1–M4, with M1 before drought, M2 after drought, M3 early recovery, and M4 recovery). (C) Example photos of the root phenotype of one genotype of the recovery field at M2 and M4. (D) Photos during recovery in the field at 25 DAS. (E) Photo of glasshouse plants 36 DAS (variety ‘ADT 12’).

To investigate variation in drought and drought recovery response, irrigated field experiments were conducted. Three consecutive field seasons were performed with 102, 89 and 120 rice accessions – primarily from among the sequenced accessions in the 3000 Rice Genomes Project (3KRG) (Wang *et al*., 2018). The trials were conducted at the Zeigler Experiment Station of the International Rice Research Institute, Los Baños, Laguna, Philippines (14°30′N, 121°15′E) in the dry seasons of 2017, 2018 and 2019. The experiments were dry-direct seeded (2 g per linear metre) into rotovated, furrowed soil (Aquandic Epiaquall) at a depth of 2–3 cm by placing 3–4 seeds every 5 cm. Two-row plots (3 m per row, 0.2 m between rows) were arranged in an augmented design with four blocks per trial. Six check varieties (Anjali, Sahbhagi dhan, Sukha dhan 4, MTU1010 and Vandana as drought-tolerant checks, and IR64 as a previous mega-variety) were replicated in each block. Basal fertilizer and sprinkler irrigation were applied 2–3 times per week until 14 d after sowing (DAS), after which the drought stress treatment was initiated by withholding of water. Rainfall during the drought stress period was 39.8 mm in 2017, 8.1 mm in 2018 and 5.6 mm in 2019. Soil moisture was monitored in each trial by tensiometers (Soil Moisture Equipment Corp.; 3–4 replicates per trial) installed at 15 cm (2018 and 2019) and 30 cm (2017–2019) depths. The drought stress treatment was ended by irrigation at 40, 34 and 35 DAS in 2017, 2018 and 2019 respectively (Fig. 1) at which time the soil water potential was −30.5 and −45.7 kPa in (2018–2019) at the soil depth of 15 cm, and −15, −19.5 and 16.7 kPa (2017, 2018 and 2019) at the soil depth of 30 cm. Soil characteristics, fieldwork activities, and grown rice accessions can be found in Supplementary Data Tables S1–S3.

### Relative growth rate

Shoots of two plants per plot were sampled twice during each trial for shoot dry weight (SDW): at the end of the drought stress treatment (M2) and after re-watering (M4) (Fig. 1) by cutting the shoots at the base. Shoot dry weights were determined after drying for several days at 60 °C, and the relative growth rate (RGR) was calculated as:


$$RGR=\frac{ln\;(SDW2)\;-ln(SDW1)}{\Delta t}$$

where Δ*t* is the number of days between sampling dates, SDW2 is the harvest weight after re-watering and SDW1 is the harvest weight at the end of drought stress. The RGR represented the g_new growth_ g_existing plant_^−1^ d^−1^ and was used as a measure of drought stress recovery. To account for spatial variation in each trial, the ls_means_ of the RGR values were determined using the *lmer* script in R v.3.4.1 (R Core Team, 2019).

### GWAS

Using data from the 3000 Rice Genome 1 Million Genome-wide association study single nucleotide polymorphism (3K RG 1M GWAS SNP) on all chromosomes (https://snp-seek.irri.org/download.zul), a genome-wide association study for RGR was analysed using all phenotyped individuals on 102, 85 and 88 lines screened based on the availability of sequence data for each accession. Using the ls_mean_ RGR values, associations for each year were run separately and analysed using a linear mixed model implemented in efficient mixed-model association (EMMA) by the R package of the Genome Association and Prediction Integrated Tool Package (GAPIT) (Zhang *et al*., 2010; Lipka *et al*., 2012). SNP marker filtering (minor allele frequency = 0.05) was performed using PLINK (Chang *et al*., 2015). The significance threshold was set to *P* < 0.0001. Co-location analysis was run by combining the GWAS results from the three years with a minimum log*P* of 3 and 100-kb co-location interval size using the *dset* and *dcast* scripts in R. To identify putative candidate genes associated with RGR, regions within 100 kb for SNPs identified from co-locating GWAS peaks between years were run in IRRI Galaxy (http://galaxy.irri.org). To determine the description/function of the genes, the genes with MSU ID were run in the Rice Genome Annotation Project (RGAP7).

### *In silico* gene expression

Gene expression analysis was performed *in silico* by accessing existing data. The openly available datasets of EMBL-EBI (Papatheodorou *et al*., 2020) were searched for loci of interest identified in the GWAS experiment.

### Recovery field experiment for root traits

A subset of lines were identified in the GWAS field experiments at IRRI, screening for contrasting growth rates and shoot dry mass in different combinations in 2017 (‘IRRI 2017’) and 2018 (‘IRRI 2018’). The second field experiment for this study had the goal to test for shoot and root responses of the ‘IRRI 2017’ and ‘IRRI 2018’ lines. During the dry season field experiment in 2018, an additional trial consisting of 20 genotypes for more elaborate root and shoot analyses was conducted. Seeds were dry seeded at a density of 2.0 g seeds m^−2^ into 2.25 m^2^ plots consisting of two rows of 3 m length × 0.25 m between rows. The plots were arranged in a randomized complete block design with four replicate plots per genotype in two treatments: a well-watered control and a drought stress treatment, which were separated by a distance of 10 m. Fertilizer and crop management practices were used per Bernier *et al.* (2007) and for the GWAS screenings described above. Both treatments were watered by sprinkler irrigation three times per week for 2 h until 14 DAS. While the well-watered control treatment was watered regularly for the duration of the experiment, the drought stress plots were dried by withholding irrigation until re-watering (Fig. 1).

A scoring system (Standard Evaluation System, SES; IRRI 2014) was used to determine the percentage of leaf dieback of the whole canopy per plot per genotype. Leaf rolling and leaf dieback were scored during the drought phase (33 DAS) and immediately after re-watering (recovery phase at 36 DAS). Leaf length was measured on the youngest and second youngest leaf on two marked plants per plot over 4 different time points (29, 34, 37, 42 DAS for the drought treatment, 29, 35, 38, 42 DAS for the control treatment). The measurement days M2 (before re-watering) and M3 (after re-watering) were delayed by 1 d in the control treatment compared to the drought stress treatment because of the amount of measurement activities in the field, and this difference was accounted for in subsequent analyses.

To characterize drought recovery, shoot and crown root sampling was performed at the end of drought stress (M2) and 1 week after re-watering (M3; Fig. 1) (Supplementary Data Table S2). Four plants per plot were selected randomly; broken, dead or diseased plants were avoided. Root crowns were excavated by digging around the plant with a trowel to ∼10 cm depth in both treatments. Root washing consisted of initial rough washing to separate the soil from the root crowns, followed by fine washing. Primary seminal and nodal axial roots were counted for total root count (Supplementary Data Fig. S1).

### Water recovery index

The WRI ranks rice lines in terms of those investing more strongly in root growth (nodal root number) or more equally towards shoot growth (leaf length) during recovery. First, the growth rate for shoots (shootGR) and roots (rootGR) is calculated based on the leaf length and crown root number at the end of drought and during recovery, divided by the number of days between the sampling points. Second, two plants were averaged per plot, followed by the average growth rates per line over four plots. It is possible to measure negative growth rates from destructive measurements; therefore, rootGR and shootGR were scaled from 1 to 100 per treatment.

The shoot and root growth rate ratio (SRGR) of each treatment was then calculated with the natural logarithm to obtain a normal distribution:


$$SRGR=ln\left(\frac{rescaled\;shootGR}{rescaled\;rootGR\;}\right)$$

After obtaining the SRGR for the drought and well-watered treatment, the SRGR well-watered values were subtracted from the SRGR drought values to obtain the WRI (Supplementary Data Table S4). Based on this calculation, the WRI was negative for our lines. The more negative WRI values, the more the line invests in crown root number, while less negative WRI values represent smaller differences between treatments. The WRI was developed to be able to compare shoot and root components of large numbers of lines within field trials and is strongly dependent on the age and growth stage of these tested lines.

### Glasshouse experiment

The third experiment was a glasshouse experiment for intensive phenotyping of two contrasting lines. The *Oryza sativa* genotypes ‘ADT 12’ and ‘ARC 18202’ (indica, upland) were chosen from the recovery field experiment as contrasting in WRI. Per genotype, 96 seeds were incubated at 50 °C for 3 d for dormancy break. After sowing in moist filter paper, two germinated seedlings were placed into soil-filled pots 1 DAS, and the second seedling was removed as soon as the first seedling was visible. The experiment was sown in two batches 2 d apart.

A sandy loam soil [‘Speyer 2.1’, LUFA Speyer (organic carbon 0.63 ± 0.07 %, nitrogen 0.05 ± 0.01 %, pH 4.7 ± 0.1); maximum water holding capacity 22.3 % (w/w) SWC ± 1.3 % (w/w) (Pflugfelder *et al*., 2017)] with a bulk density of 1.17 g cm^−3^ was used. The soil was air-dried for >7 d, dried at 60 °C for >1 d and watered to 5 % (w/w) before filling pots each with 2453 g soil and 390 g water (=16.725 w/w water content = 75 % water holding capacity) (Supplementary Data Fig. S2). A fleece inlay and a surrounding, closed plastic bag prevented evaporation in the first days. The pots were arranged in eight randomized blocks, split in equal parts drought and control.

The glasshouse conditions were 26 °C during the day and 20 °C at night. Relative humidity was adjusted to 80 %, aided by a humidifier consisting of an ultrasound fogger (Techsin 19–12, China) floating on water in a barrel and fogging 2.5 L water per hour. The humidified air was directed to the plants through a hose with evenly distributed holes for 45 min within 1 h for 12 h daily. The pots were watered to their initial weight every other day, and leaf length was measured (tip to base). Drought was applied by withholding water until plants expressed leaf growth <1 cm per day for >2 d, and leaf rolling was visible in each genotype.

### Destructive plant harvest and measurements

Four destructive harvests were performed with a sample size of six plants each: 14 DAS (before drought; M1), 29 DAS (after drought; M2), 30 DAS (1 d after re-watering; M3) and 36 DAS (7 d after re-watering; M4; see Fig. 1). At M1–M4, leaf length and area (LI-3100C Area Meter; LI-COR, USA) were measured, followed by drying leaves and stems for >7 d at 60 °C to determine shoot dry weight. The soil was removed by running tap water, and washed roots were photographed.

Neutral Red was employed as a stain for living cells (Ehara *et al*., 1996; Dubrovsky *et al*., 2006). Neutral Red staining was performed on the longest seminal root (3 cm from the tip) and, when present, on the second longest seminal root and the longest crown root. The remaining root system was stored in 50 % EtOH at 4 °C. Roots were incubated for 24 h in Neutral Red staining solution (0.0004 mg mL^–1^ in 0.1× PBS pH 7.4) followed by washing and storing (0.1× PBS pH 7.4) at 4 °C until evaluation. Transmitted light was used and three random photos per root were taken for staining scoring using a stereo microscope (MZ12.5, Leica). A scoring system with six intensities was developed per root class: main (seminal/crown root), S-type LR, L-type LR and 2nd-order LR (Supplementary Data Fig. S3), and root diameter was determined using Image J (Fiji) to allow root type-dependent scanning.

A flatbed scanner (Expression 10000 XL, Epson, resolution 600 dpi) with WinRHIZO (Pro 2020 Regent Instruments, Canada) was used for root scanning of the entire root system with the diameter classes 0–0.15 mm (S-type LRs), 0.15–0.37 mm (L-type LRs), and >0.37 mm (seminal and crown roots). In addition, a link analysis was performed to determine the average length of S-type LRs with the ‘External-Internal’ count. As WinRHIZO cannot differentiate between first-order (branching off seminal or crown roots) and second-order (branching off L-type LRs) S-type LRs, their length was determined in staining images from M2 using ImageJ (Fiji).

Note that until M1 the plants experienced identical conditions in each treatment.

### Statistical analyses

To account for spatial variation in each trial, the lsmeans of the RGR values were determined using the lmer script in R v.3.4.1 (R Core Team, 2019). For the recovery field experiment, all data were analysed using Microsoft Excel 2016 (Microsoft, Redmond, WA, USA) and R Studio v.1.0 (R Studio Inc., Boston, MA, USA). An ANOVA (*aov* script) was carried out.

For the glasshouse experiment, all data were analysed using Microsoft Excel 2016 and R Studio v.2023.03.1. A Type II ANOVA (*aov* script, car package) was carried out with Tukey’s honestly significant difference test as a post-hoc test.

## RESULTS

### Genetic association of shoot growth recovery

**Fig. 2.**
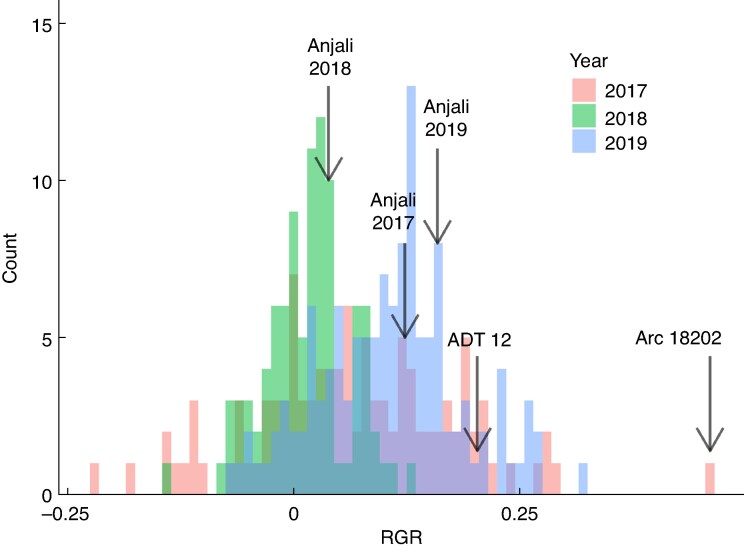
Results of the genome-wide association study of three field trials. Distribution of lsmeans of relative growth rates (RGR; g_new growth_ g_existing plant_^−1^ d^−1^) among the three separate sets of accessions grown in field trials in 2017 (red), 2018 (green) and 2019 (blue). Arrows indicate the RGR lsmean values of ‘Anjali’, which was the check variety with the highest values in all three trials, and ‘ADT 12’ and ‘ARC 18202’.

To characterize genetic diversity in rice drought recovery ability, 100 different rice genotypes from the 3KRG per three dry seasons were screened as part of an effort to identify new donors for crossing in the IRRI breeding programmes. SDW measurements before and after re-watering were taken to calculate the relative growth rate (relGR) of SDW as an indicator of drought recovery. The median RGR lsmean value was 0.064 in 2017, 0.019 in 2018 and 0.111 in 2019 (Fig. 2). ‘Anjali’ showed the highest RGR lsmeans of the six check varieties in all three trials (Fig. 2). Compared to ‘Anjali’, there were 35 accessions with higher RGR lsmean values in 2017, 25 in 2018 and 26 in 2019. A GWAS was performed on the RGR values from individual years, and GWAS peaks were low/non-significant (maximum –log_10_  *P*-values were 5, 4 and 4 in 2017, 2018 and 2019, respectively; Fig. 2). The highest peaks were observed in 2017 on chromosomes 1 and 11, on chromosome 6 in 2018, and on chromosomes 2 and 11 in 2019 (Supplementary Data Fig. S4). A co-location analysis identified peaks on chromosomes 3, 6 and 10 that co-located between 2017 and 2018, chromosomes 8 and 11 that co-located between 2017 and 2019, and chromosome 5 that co-located between 2018 and 2019 (Supplementary Data Tables S5, S6 and S7). Candidate genes from the peaks on chromosomes 3, 5, 6, 8, 10 and 11 were also identified (Supplementary Data Tables S5, S6 and S7).

These potential candidate genes indicated by GWAS based on RGR were validated *in silico* (see Methods). One of these, *Sucrose synthase 1* (*OsSUS1*; *LOC_Os03g28330*), has been connected to phosphorus starvation (Yang *et al*., 2019), salt stress (Saha *et al*., 2020), and grain quality and starch synthesis (Liu *et al*., 2022). Two identified loci were connected to salt stress [*LOC_Os03g37260* (Rohila *et al*., 2019); and *LOC_OS03g37290* (Zhou *et al*., 2016)], while two others were shown to be involved in rice root development [*LOC_0s03g31880*, *OsSHR2* (Liu *et al*., 2023); *LOC_Os11g43320*, *OsRL11.1* (Zhao *et al*., 2018)]. Additionally, several loci were identified in previous studies to be involved in abiotic stresses such as heat [*LOC_Os10g20990* (Peng *et al*., 2020)], phosphorus starvation [*LOC_Os10g20990* (Cai *et al*., 2012); *LOC_OS11g43760* (Secco *et al*., 2013)] and drought [*LOC_Os11g43790* (Kumar *et al*., 2022)].

### Diverse shoot and root responses during recovery leading to the WRI

Since the GWAS trial was based solely on shoot variation, an additional field trial with 20 selected genotypes was conducted allowing multi-time point non-invasive (leaf length) and invasive (crown root number) sampling to capture the shoot and root dynamics of recovery after drought. Leaf rolling and leaf dieback in the drought treatment were consistent with known rice drought responses (Supplementary Data Fig. S5). Following re-watering, the plants recovered quickly by leaf un-rolling. Varying effects of treatment and genotype were found for leaf length and crown root number (Supplementary Data Figs S6 and S7).

**Fig. 3.**
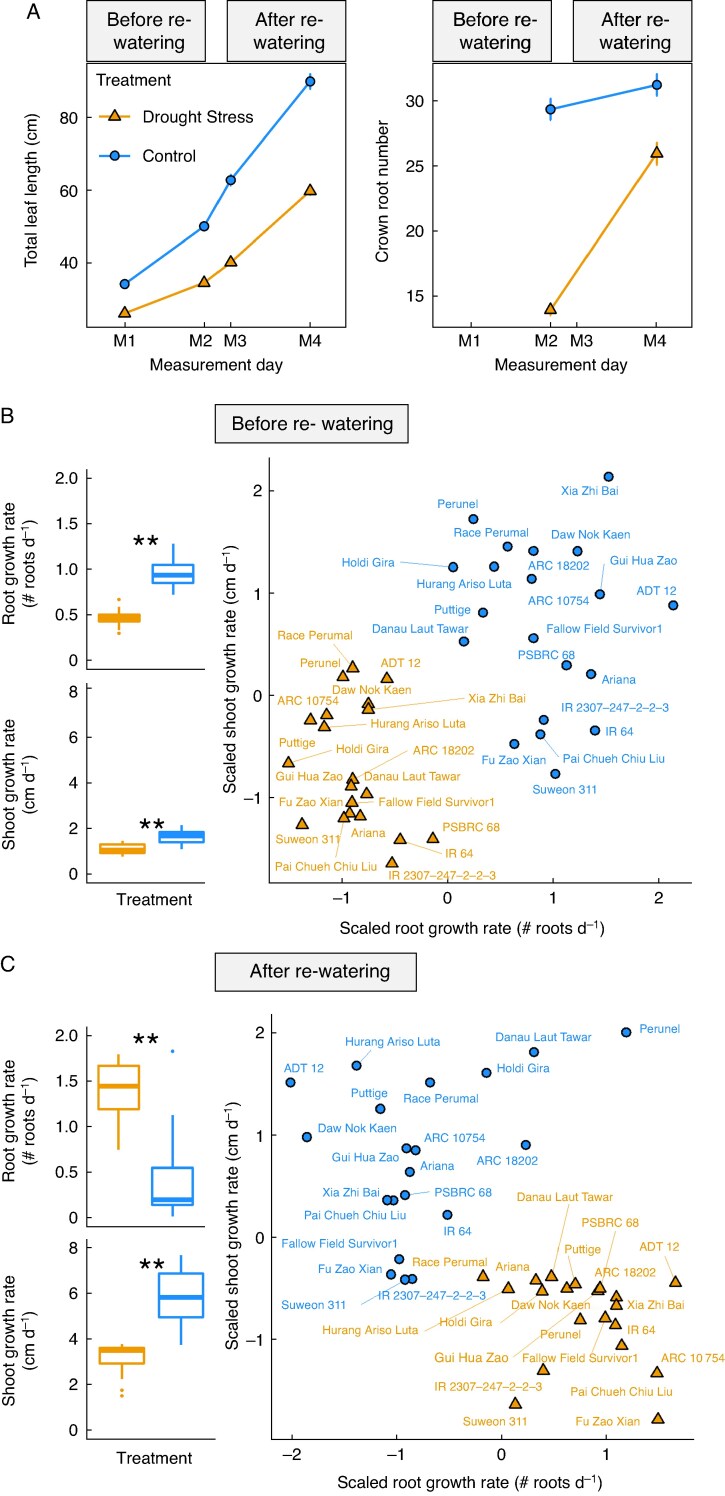
Root and shoot growth increase differently under drought. Leaf length and crown root number were determined in the recovery field experiment for 20 genotypes with drought treatment (orange) or well-watered (blue). Line plots show the mean across all genotypes ±SE (*n* = 160 for total leaf length; *n* = 320 for crown root numbers) over time (A). Calculated shoot and root growth rate over all genotypes and (left) as scatter plots per genotype (right) before (B) and after (C) re-watering. The growth rates were scaled to a mean value of zero to visualize differences in genotypic growth patterns. A one-sample *t*-test was used (*P < 0.05, **P < 0.01).

Destructively harvested plants allowed several observations of root development, with crown root number (CRN) increasing over time (Supplementary Data Fig. S7). Thirteen out of the 20 genotypes had similar average CRNs after drought and throughout recovery (M4) compared to the well-watered control plants (Supplementary Data Fig. S7). On average, well-watered plants had greater leaf length and a higher number of crown roots than the drought-treated plants (Fig. 3A). The root growth rate was calculated across all genotypes before and after re-watering. Plants under drought stress had lower root growth rate (rootGR) before re-watering (Fig. 3B) compared to well-watered control plants (*t*-test, *P* < 0.001), while higher rootGRs (Fig. 3C) were found after re-watering (*t*-test, *P* < 0.001). In contrast to the roots, the shoot growth rates (shootGRs) in the drought-stressed plants both before and after re-watering were lower than in well-watered plants (Fig. 3B and C; *P* < 0.001).

**Fig. 4.**
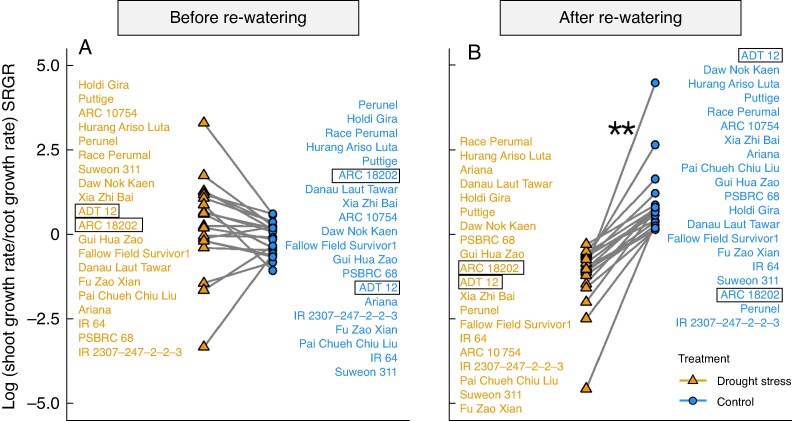
Increased root compared to shoot growth following re-watering after drought. Shoot and root growth rate ratio (SRGR; ln-transformed ratio of SGR and RGR) in drought (orange) and control (blue) plants before (A) and after re-watering (B) across all 20 genotypes in the recovery field experiment. Null values represent no difference between tissues. Connected lines represent one genotype. A one-sample *t*-test was used (*P < 0.05, **P < 0.01). Framed genotypes were selected for further glasshouse studies.

To gain further insight into whole-plant responses to drought recovery, shoot and root development were combined into a new value, the WRI. For that, the shoot and root growth rate ratio (SRGR) was calculated (see Methods). The SRGRs in the drought and well-watered plants were similar (log-transformed, *t* = 1.204, *P* = 0.241, Fig. 4A) before re-watering. In contrast, after re-watering, the SRGRs of drought-treated plants were significantly lower compared to the well-watered plants (log-transformed, *t* = −6.737, *P* <0.001, Fig. 4B), indicating higher allocation to root than shoot growth when water was resupplied (referred to as WRI). ‘ADT 12’ and ‘Fu Zao Xian’ had the lowest WRIs (low ratio of shoots to roots in the drought compared to well-watered control), while ‘ARC 18202’ and ‘Danau Laut Tawar’ had the highest WRI (higher ratio of shoots to roots in the drought treatment compared to well-watered plants).

### Lateral roots are instrumental to drought recovery

**Fig. 5.**
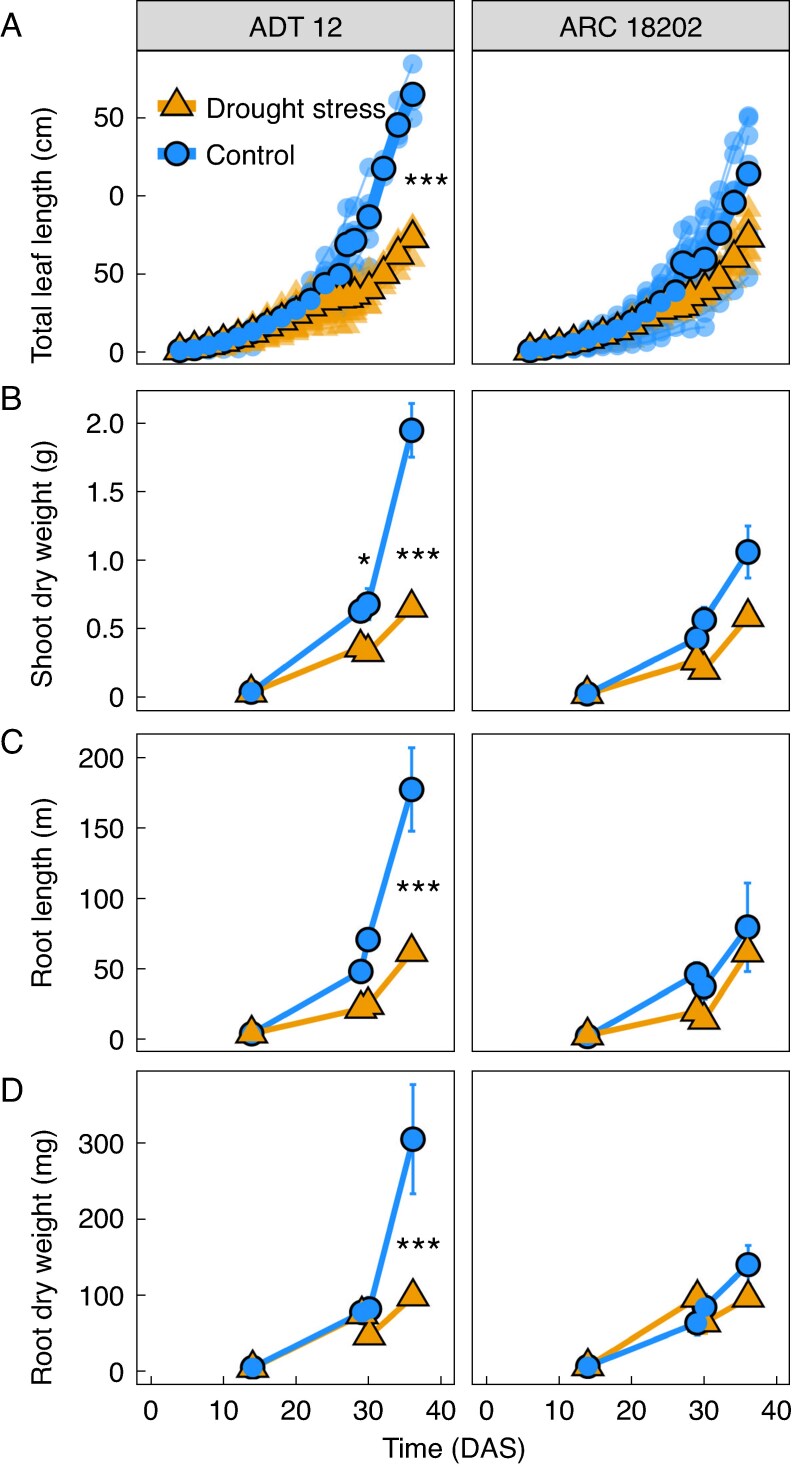
Dynamic plant development through drought and recovery. Total leaf length (A), shoot dry weight (B), root length (C) and root dry weight (D) of drought-stressed (orange) and well-watered control (blue) plants measured at M1 (before drought), M2 (after drought), M3 (1 d after re-watering) and M4 (7 d after re-watering) in the glasshouse experiment. Depicted as single plants (transparent circles) and mean values (full circles, A) or as mean ± SE (B–D). For A and B *n* = 6, and for C and D *n* = 3. Statistical analysis was performed between treatments as Type-II ANOVA with a Tukey HSD test (***P < 0.001, **P < 0.01, *P < 0.05). For plot A only the last day of measurement was tested for differences.

To gain a more detailed understanding of root and shoot development during drought and recovery, an in-depth glasshouse experiment with two WRI-contrasting lines, ‘ADT 12’ and ‘ARC 18202’, was conducted (Fig. 1). These lines differed in the influence of drought on their root-to-shoot ratios. Well-watered plants exhibited longer leaf lengths and higher shoot dry weight compared to their drought-stressed counterparts. While ‘ADT 12’ leaves were longer and had more biomass in the well-watered condition compared to drought, ‘ARC 18202’ had a stable leaf length as well as dry weight under drought compared to well-watered conditions (Fig. 5A and B). Root length and root dry weight developed differently for both lines, with a higher root length and root dry weight after re-watering for ‘ADT 12’ under well-watered conditions, whereas the root development of ‘ARC 18202’ did not differ after re-watering between treatments (Fig. 5C and D). Drought inhibited crown root elongation, not formation, for both genotypes; re-watering promoted crown root outgrowth. Under field conditions, comparable observations were made – crown root length was inhibited by drought (Supplementary Data Fig. S1) while crown root number was increased under re-watering conditions (Supplementary Data Fig. S7).

**Fig. 6.**
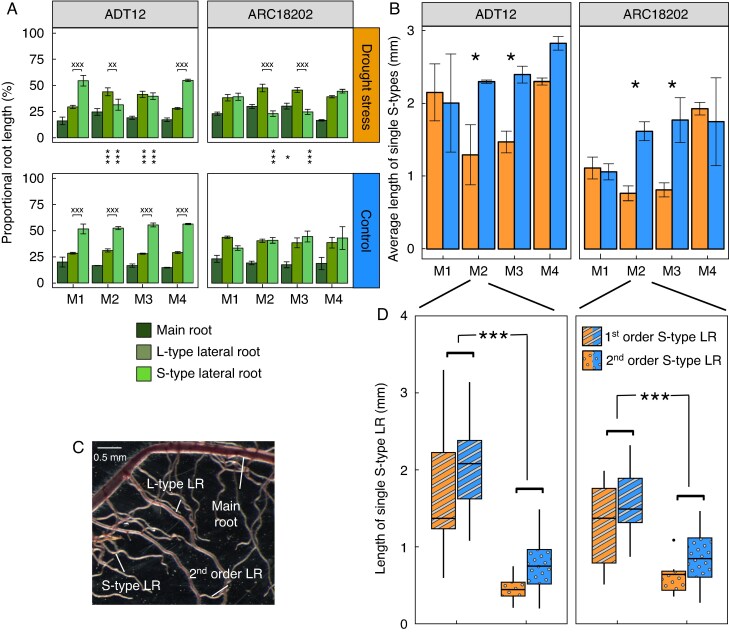
Root type length was selectively affected by drought stress. Total root length of the glasshouse experiment was determined by WinRhizo scanning and separated per root type between main roots (seminal and crown roots), L-type LRs and S-type LRs (example image of root types in C) at the four measurement days as mean ± SD (*n* = 3) (A). Different letters indicate significant difference within a genotype per treatment determined by ANOVA and the Tukey test (*P* < 0.05). Significant differences between treatments per genotype are indicated by asterisks. Average length of single S-type LRs determined by WinRhizo link analysis (B) and length of single S-type LRs measured using a stereo microscope after drought (D). Depicted are mean ± SD (*n* = 7–12). A Type-II ANOVA followed by a Tukey HSD test was used to test the differences within the genotypes (***P < 0.001, **P < 0.01, *P < 0.05, ^xxx^*P* < 0.001, ^xx^*P* < 0.01, ^x^*P* < 0.05).

To obtain a more detailed dynamic view of the root system, three types of roots were separated: (1) axial roots (crown and seminal roots, diameter > 370 µm); (2) L-type LRs (diameter 150–370 µm); and (3) S-type LRs (diameter < 150 µm) identifying root length per root type. Total root length and root proportion were calculated. While well-watered plants did not show proportional length differences in root type over time, under drought stress proportionally more L-type LR length was developed and less S-type LR length (Fig. 6A). Notably, 7 d after re-watering (M4), the root length proportion of every root type in the drought treatment was comparable to that of the well-watered control plants. Overall, ‘ADT 12’ had a higher proportion of S-type LRs (>50 %) than L-type LRs (∼28 %) during establishment (M1, before drought stress) compared to ‘ARC 18202’ (both ∼38 %). Briefly both lines increased the proportion of L-type LRs under drought, but for ‘ADT 12’ this change was more intense and significant. ‘ARC 18202’ initially had an equal proportion of S-type LRs and L-type LRs and therefore had only a minor change under drought. Moreover, both lines had stable proportions of LRs under well-watered conditions over time.

The average length of individual S-type LRs was determined by WINRHIZO link analysis (1st- and 2nd-order S-type LRs combined, see Methods) and increased over time in well-watered plants, whereas it decreased during drought (Fig. 6B). For ‘ARC 18202’ and ‘ADT 12’, this decrease was compensated for after recovery. To test if the decrease was due to death or reduced growth, we tested the longevity of S-type LRs and measured their length. In both genotypes, 1st-order S-type LRs grew longer compared to 2nd-order S-type LRs (branched off L-type LRs), and drought stress did not influence the length of either (Fig. 6D).

Longevity was scored to distinguish living and dead root parts. Both genotypes had similar staining scores in the well-watered condition (medium to high proportion of longevity) (Supplementary Data Fig. S9). At the end of the drought stress (M2) and directly after 1 d of re-watering (M3), both genotypes had more living root tissue compared to well-watered plants, while longevity decreased in both treatments at the end of recovery (M4). Parental roots decreased their proportional living root length over time, while all LR classes had higher longevity values when experiencing drought (M2) or shortly after (M3) in both genotypes (Supplementary Data Fig. S9). Taken together, drought stress reduced the average S-type LR length within the root systems mainly by increasing the number of 2nd-order S-type LRs (S-type LRs branching from L-type LR) and sustaining higher longevity of LRs.

## DISCUSSION

Fluctuating soil moisture status will be a critical problem for food security in the future, leading to an increased risk of drought in different global areas, including Asia (Li *et al*., 2024). Therefore, research on drought and drought recovery is and will be of growing importance in the coming years, especially in developing countries where rice is a staple crop that needs to thrive under more extreme conditions due to changing climates and limited water supply. The present study compared and screened rice diversity panels with contrasting shoot-to-root development under drought and recovery under field conditions. The contrasting lines ‘ADT 12’ and ‘ARC 18202’ were intensively investigated in a drought and re-watering experiment in glasshouse pots. ‘ADT 12’, the larger genotype, reacted with stronger growth inhibition to drought, whereas ‘ARC 18202’, the smaller genotype, was more resistant. Drought caused smaller changes in the root type proportions of the smaller genotype, which could represent a benefit under drought. However, detailed root investigations yielded the same general trend of predominance of L-type LRs under drought and S-type LRs without drought.

Associating relative shoot growth to genomic differences yielded only non-significant peaks, mostly due to high phenotypic variation in the field trials. Yet the identified candidate genes here were previously connected to heat stress, phosphorus starvation and drought. Further genetic studies and validation studies are needed to identify genes that can be used for breeding programmes to improve future drought recovery scenarios. Characterization of existing breeding pools not only for shoot response, but also root traits and genetics, is needed to understand how the insights on drought recovery revealed by this study can be most effectively applied. Although root studies are labour-intensive and more difficult due to the ‘hidden half’ phenotyping, they will be crucially important for further research linking phenotype to genotype (Teramoto and Uga, 2022).

**Fig. 7.**
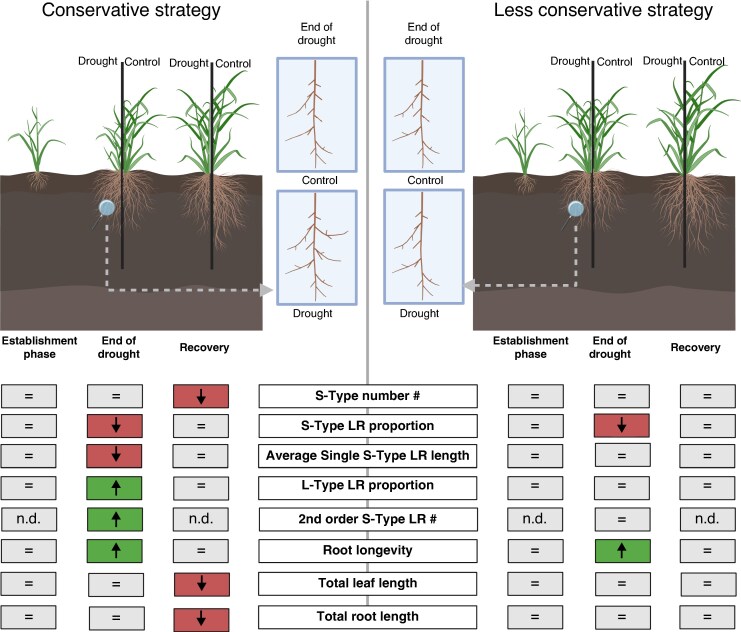
Conservative and less conservative strategies in drought stress involve LRs in rice. Schematic overview of the development of conservative (left) or less conservative (right) rice plants when faced with drought stress and recovery (top). Progress of traits at establishment phase (M1), drought (M2) and recovery (M4) for both strategies as the difference of drought to control (bottom). Green indicates an increase compared to the control, red a decrease, = values not significantly different between drought and control; n.d. not determined. Created in BioRender. Bochmann, H. (2025) https://BioRender.com/j08j289.

To widen our analyses of rice drought recovery, root responses were analysed as well as shoot growth. Nodal root number was overall suppressed under drought stress in our study, but the growth rate expressed as nodal root number per day was higher in the drought stress compared to the well-watered control group after re-watering, indicating a recovery effect of nodal root growth in response to water resupply. Nodal root development is known to be more susceptible to drought stress than LR formation, but the intensity depends on the genotype (Kameoka *et al*., 2016). The shoot growth represented by leaf length is widely known to be suppressed under drought (as reviewed in Jarin *et al*., 2024). In our study, a significant reduction of leaf length elongation was observed, with less growth under drought and recovery compared to the well-watered plants. Therefore, the WRI, calculated based on leaf length and nodal root number, can be used to compare rice lines in one experiment under drought and re-watering response, combining the traits of shoot-to-root ratio for the well-watered plants compared to that under drought stress. The WRI as a normalized root-to-shoot ratio is a measurement capable of detecting contrasting drought recovery strategies, which can be used to screen lines for variable drought stress scenarios. The WRI identified different growth allocations, indicating different strategies in drought stress response. Growth of ‘ADT 12’, the larger genotype, was strongly suppressed under drought, but also increased strongly after re-watering, which we determined as a ‘conservative’ strategy (Fig. 7). Growth of the smaller genotype, ‘ARC 18202’, was less suppressed by limited water supply and therefore little effect of re-watering was measured – this was determined as a ‘less-conservative’ strategy. The conservative strategy leads to a greater root length for water acquisition after drought while shoot growth is resumed later. Moreover, due to the shift in root growth to shoot growth, water uptake per individual unit root area is reduced and prevents faster drying during a repeated drought scenario. More root length in deeper soil is favourable in water-limited environments, but this advantage depends on shoots (Kato *et al*., 2007). The trade-off is a higher cost for root system growth, reducing energy available for above-ground biomass investment. ‘ARC 18202’ showed a contrasting development and WRI value, indicating a more balanced investment in shoot and root growth. This less-conservative strategy can be interpreted as drought resistance in our experiments as ‘ARC 18202’ growth was similar between treatments. The maintenance of growth and root-to-shoot ratio was beneficial under the fluctuating water supply in our experiments, yet it may be less or not beneficial in other drought scenarios. In accordance with other studies (Fonta *et al*., 2022*b*; Vadez *et al*., 2024), further research is necessary on which strategy is needed for specific drought environments and conditions.

Our intensive phenotyping in the glasshouse revealed partly overlapping, but also contrasting, drought responses in LR formation under drought between the two tested lines. Previously, it was shown that higher root plasticity contributed to higher drought tolerance, including deeper L-type LR formation (Fonta *et al*., 2022*a*). In our study, we found that both rice lines increased L-type LR growth during drought, including a higher amount of short 2nd-order S-type LRs branching off L-type LRs. In Egyptian rice lines, L-type LR and S-type LR length also increased by drought treatment (Hazman and Brown, 2018), but a re-watering period was not evaluated. In our study, 1 week after re-watering, both lines showed the same root type proportion as their well-watered control plants. This is a strong indication of plasticity in root system formation that is driven by both genetic features as well as environmental conditions. The well-watered root system seems to be determined by genetics, whereas the drought treatment induced phenotypic changes, which were reversed after rewatering. This impressive finding of the reversibility of this drought-adapted root system should be investigated further, especially with regard to the increased need for drought recovery research in the future. Both lines had a comparable root length at the end of drought stress, with different proportions of root types. Influence of drought stress on LRs has been addressed previously (De Bauw *et al*., 2020; Watanabe *et al*., 2020), but the root type proportions of well-watered, drought and re-watered phenotypes is an equally important area of research and should be expanded in the future, adding to the research done in this field (Bañoc *et al*., 2000; Suralta *et al*., 2008).

## CONCLUSION

Recovery from drought is an increasingly researched area and holds large agricultural importance and relevance for future food security. Our experiments yielded contrasting root and shoot growth responses summarized by the WRI and distinguishing conservative from less-conservative strategies. With unlimited water supply, the conservative genotypes would outperform the less conservative ones. However, the ability of the less conservative genotypes to keep up their growth during drought highlights their robustness and makes them ideal candidates for investigating the adaptive mechanisms and genomic loci linked to improved drought resilience. Ideally, these genetic components would be bred into conservative, high-yielding genotypes to obtain new lines more tolerant to drought periods, as suggested recently (Heredia *et al*., 2022). Interestingly, recovery not only allowed shoot and root regrowth but also enabled both genotypes to resume the S-type:L-type LR proportions of a well-watered root system. Despite the differing drought recovery strategies observed in the field that were reflected by shoot growth, the rice genotypes studied here exhibited a more similar response in their root system plasticity response to drought. This interplay between genetic and environmental adaptations should be investigated in the future.

## Supplementary Material

mcaf173_Supplementary_Data

## SUPPLEMENTARY MATERIAL

Supplementary data are available at *Annals of Botany* online and consist of the following. Table S1: Soil nutrient analysis at upland UI field site. Table S2: Activity protocol of field experiment for the end of April until early May 2018 at IRRI, Block UI3 upland area. Table S3: Lines used in the GWAS. Table S4: Calculation of the WRI based on difference of shoot and root growth rate ratio (SRGR) of drought treatment and controls of all genotypes. Table S5: GWAS results 2017. Table S6: GWAS results 2018. Table S7: GWAS results 2019. Figure S1: Example of crown roots sampling. Figure S2: Dynamic water content over the experimental timeframe. Figure S3: Neutral Red staining references. Figure S4: GWAS peaks 2017–2019. Figure S5: Leaf rolling responses to drought per genotype. Figure S6: Genotypic diversity in shoot responses to re-watering after drought. Figure S7: Genotypic diversity in root responses to re-watering after drought. Figure S8: Count of S-type LRs. Figure S9: Root staining.
