# Supplementary material for: Rice cycles between drought and well-watered-adapted phenotypes by changing lateral root formation
Source: Ann Bot. 2025 Aug 12;136(5-6):1265–77. doi: 10.1093/aob/mcaf173 (PMC12682850; doi:10.1093/aob/mcaf173)
Supplement: mcaf173_Supplementary_Data [file mcaf173_supplementary_data.pdf]

Supplementary Information Table 1

Soil Nutrient Analysis at Upland UI field site.

The soil was analysed at IRRI Analytical Service Laboratory. Soil samples were taken at 0-15 cm depth for nutrient analyses.

Avail P-O (mg/kg)	Kj N (%)	Org C (%)	pH (H2O)	Exch K (meq/100g)	Exch Mg (meq/100g)	Exch Ca (meq/100g)	Clay P (%)	Sand P (%)	Silt P (%)
44	0.1095	0.953	6.65	1.51	9.59	15.55	32.5	17.5	50

Supplementary Information Table 2

Activity protocol of field Experiment for the end of April until early May 2018 at IRRI, Block UI3 upland area.

Date	DAS	Activity
21-Mar	0	Direct sowing
2-Apr	12	Basal fertilizer
4-Apr	14	Last watering
16-Apr	26	Weeding
19-Apr	29	First measurement day (M1)
25-Apr/ 26-Apr	35/36	Second measurement day (M2)
26-Apr	36	Re-watering
27-Apr/ 28-Apr	37/38	Third measurement day (M3)
2-May	42	Fourth measurement day (M4)

Supplementary Information Table 3
GWAS lines

Genetic_Stock_varname	Genetic_Stock_ Accession	DNA Unique ID	Experiment Year
Vandana	IRGC 136064	CX236	2017
IR 2344-P1 PB-9-3-2B::IRGC 39317-C1	IRGC 122093	IRIS_313-7690	2017
IR 62266-42-6-2::IRGC 117397-1	IRGC 117597	IRIS_313-8232	2017
JAGLI BORO::IRGC 27516-2	IRGC 121233	IRIS_313-8252	2017
URAIBOOL::IRGC 52785-1	IRGC 126289	IRIS_313-8305	2017
BAT DO::IRGC 7014-1	IRGC 121599	IRIS_313-8341	2017
KURULU WEE (WHITE)::IRGC 66518-1	IRGC 121029	IRIS_313-8342	2017
ARC 10812::IRGC 21074-1	IRGC 126173	IRIS_313-8386	2017
SUFAID 246::IRGC 28303-1	IRGC 121128	IRIS_313-8390	2017
KHARSU 80::IRGC 28016-1	IRGC 121022	IRIS_313-8398	2017
JIN JUN DAO::IRGC 59710-1	IRGC 125613	IRIS_313-8405	2017
FEI GAI 122::IRGC 63599-1	IRGC 125609	IRIS_313-8412	2017
ARC 18202::IRGC 42328-1	IRGC 126175	IRIS_313-8414	2017
CHANG LE SAN SHU ZAO::IRGC 63561-1	IRGC 126115	IRIS_313-8433	2017
UPRH 233::IRGC 61667-1	IRGC 125627	IRIS_313-8435	2017
ARC 18597::IRGC 43299-1	IRGC 126043	IRIS_313-8453	2017
DAA MANSA::IRGC 67559-1	IRGC 126196	IRIS_313-8514	2017
DHANE BURWA::IRGC 10105-1	IRGC 126199	IRIS_313-8530	2017
KEERIPALA CHILL PADDY::IRGC 49790-1	IRGC 125615	IRIS_313-8559	2017
ARC 11901::IRGC 21727-1	IRGC 127033	IRIS_313-8585	2017
PAI YI PING::IRGC 1368-1	IRGC 126122	IRIS_313-8645	2017
PERUNEL::IRGC 63113-1	IRGC 125621	IRIS_313-8647	2017
YEBAWYIN::IRGC 33885-1	IRGC 125628	IRIS_313-8697	2017
PULUT BARAYA::IRGC 27393-1	IRGC 126262	IRIS_313-8725	2017
NIBARI::IRGC 67742-1	IRGC 126119	IRIS_313-8731	2017
DUDH KADAR::IRGC 67707-1	IRGC 125607	IRIS_313-8796	2017
LU MAO ZHAN::IRGC 68159-1	IRGC 125829	IRIS_313-8889	2017
BPI 76 NON SENSITIVE (GREEN)::IRGC 979	IRGC 120902	IRIS_313-8903	2017
E 2040::IRGC 67968-1	IRGC 125731	IRIS_313-8914	2017
PUTTIGE::IRGC 52588-1	IRGC 125879	IRIS_313-8921	2017
KUTTA::IRGC 52184-1	IRGC 125815	IRIS_313-8924	2017
FU ZAO XIAN::IRGC 63619-1	IRGC 125744	IRIS_313-8940	2017
ARC 11524::IRGC 42672-1	IRGC 125641	IRIS_313-8946	2017
BATHURI::IRGC 25838-1	IRGC 120887	IRIS_313-8963	2017
ARC 12576::IRGC 22163-1	IRGC 125645	IRIS_313-8967	2017
ARC 18112::IRGC 42274-1	IRGC 125655	IRIS_313-8982	2017
ARC 10754::IRGC 12603-1	IRGC 125637	IRIS_313-8986	2017
CR 60-10::IRGC 15777-1	IRGC 125715	IRIS_313-9023	2017
PAI CHUEH CHIU LIU::IRGC 34259-1	IRGC 125865	IRIS_313-9065	2017
XIA ZHI BAI::IRGC 53437-1	IRGC 125940	IRIS_313-9111	2017
XI GAN JING REN::IRGC 60035-1	IRGC 126014	IRIS_313-9184	2017
CHI SHENG TAO::IRGC 4606-1	IRGC 125704	IRIS_313-9204	2017

YONG JIN ZAO 3::IRGC 70441-1	IRGC 125946	IRIS_313-9253	2017
TAK SUFAID::IRGC 73127-1	IRGC 121134	IRIS_313-9283	2017
PALEPYU::IRGC 33549-1	IRGC 125866	IRIS_313-9285	2017
PARA NELLU::IRGC 50009-1	IRGC 125868	IRIS_313-9351	2017
CHANDARHAT::IRGC 25845-1	IRGC 121605	IRIS_313-9368	2017
ARC 18092::IRGC 42256-1	IRGC 125654	IRIS_313-9427	2017
E 2024::IRGC 67958-1	IRGC 125730	IRIS_313-9482	2017
NCS 237::IRGC 62202-1	IRGC 125853	IRIS_313-9492	2017
ARC 11857::IRGC 40972-1	IRGC 125643	IRIS_313-9560	2017
NX 3533::IRGC 63796-1	IRGC 125859	IRIS_313-9570	2017
ASU::IRGC 62154-1	IRGC 125658	IRIS_313-9572	2017
RPA 5929 (K 45)::IRGC 33963-1	IRGC 125890	IRIS_313-9604	2017
ARC 10594::IRGC 12524-1	IRGC 125636	IRIS_313-9609	2017
WANGA BARUGULU::IRGC 52261-1	IRGC 125935	IRIS_313-9611	2017
TSAI YUAN CHON::IRGC 126-1	IRGC 125928	IRIS_313-9708	2017
BA SHI ZAO::IRGC 67903-1	IRGC 125675	IRIS_313-9730	2017
I KUNG PAO::IRGC 114-1	IRGC 125770	IRIS_313-9758	2017
GENIT::IRGC 3272-1	IRGC 125751	IRIS_313-9778	2017
CRILLO LA FRIA::IRGC 10793-1	IRGC 125716	IRIS_313-9822	2017
MILYANG 30::IRGC 46977-1	IRGC 125839	IRIS_313-9925	2017
RACE PERUMAL::IRGC 55347-1	IRGC 125881	IRIS_313-9970	2017
SSANGDUJO::IRGC 55632-1	IRGC 125906	IRIS_313-9976	2017
MELEKE::IRGC 56823-1	IRGC 125838	IRIS_313-9989	2017
MILYANG 77::IRGC 69340-1	IRGC 125840	IRIS_313-10040	2017
SAN SHIH TSI::IRGC 1038-1	IRGC 125897	IRIS_313-10129	2017
CAUVERY::IRGC 45255-1	IRGC 125695	IRIS_313-10148	2017
HE GU TSAO::IRGC 51302-1	IRGC 125760	IRIS_313-10154	2017
IR 13429-109-2-2-1::IRGC 63491-1	IRGC 125773	IRIS_313-10167	2017
DA GANG ZHAN::IRGC 67103-1	IRGC 125719	IRIS_313-10177	2017
GAO JIAO BAI::IRGC 68047-1	IRGC 125748	IRIS_313-10178	2017
GUI HUA ZAO::IRGC 68060-1	IRGC 125755	IRIS_313-10179	2017
DA NUO (ZHAN)::IRGC 72025-1	IRGC 125723	IRIS_313-10189	2017
MIN KE ZHAN::IRGC 72230-1	IRGC 125841	IRIS_313-10191	2017
BA BAI GU::IRGC 79580-1	IRGC 125663	IRIS_313-10221	2017
TONG GU HONG::IRGC 81026-1	IRGC 125925	IRIS_313-10226	2017
PSBRC 68::IRGC 99711-1	IRGC 125874	IRIS_313-10235	2017
ICTA CRISPO 38::IRGC 116994-1	IRGC 125967	IRIS_313-10274	2017
CAMPONI::IRGC 116963-1	IRGC 125956	IRIS_313-10325	2017
CSR-90 IR-2::IRGC 117327-1	IRGC 120921	IRIS_313-10349	2017
IR 80310-12-B-1-3-B::IRGC 117307-1	IRGC 125986	IRIS_313-10394	2017
IR 80340-23-B-12-6-B::IRGC 117309-1	IRGC 125987	IRIS_313-10396	2017
IRGA 318-11-6-9-2B::IRGC 117339-1	IRGC 120991	IRIS_313-10397	2017
IRGA 370-38-1-1F-C4-2::IRGC 117342-1	IRGC 125988	IRIS_313-10400	2017
IRGA 370-42-1-1F-C-1::IRGC 117343-1	IRGC 125989	IRIS_313-10401	2017
IRGA 659-1-2-2-2::IRGC 117345-1	IRGC 120993	IRIS_313-10403	2017
ADT 12::IRGC 6254-1	IRGC 127107	IRIS_313-10527	2017
AUS 171::IRGC 29004-1	IRGC 127178	IRIS_313-11048	2017

AUS 219::IRGC 29031-1	IRGC 127179	IRIS_313-11049	2017
AUS 233::IRGC 29036-1	IRGC 127180	IRIS_313-11050	2017
AUS 278::IRGC 29068-1	IRGC 127182	IRIS_313-11052	2017
AUS 295::IRGC 29083-1	IRGC 127184	IRIS_313-11054	2017
AUS 301::IRGC 29089-1	IRGC 127186	IRIS_313-11056	2017
AUS 308::IRGC 29096-1	IRGC 127187	IRIS_313-11057	2017
AUS 329::IRGC 29116-1	IRGC 127188	IRIS_313-11058	2017
AUS 344::IRGC 29131-1	IRGC 127189	IRIS_313-11059	2017
BAK TULSI::IRGC 34831-1	IRGC 127205	IRIS_313-11164	2017
AUS PADDY (RED)::IRGC 44978-1	IRGC 127192	IRIS_313-11348	2017
CN 44-40-7::IRGC 45368-1	IRGC 127286	IRIS_313-11355	2017
CUN GU NUO::IRGC 63576-1	IRGC 127288	IRIS_313-11664	2017
CHIAYI WU-K'O::IRGC 64974-1	IRGC 127273	IRIS_313-11692	2017
G Zhenshan 97B	NA	B156	2018
Minghui 63	NA	CX145	2018
Vandana	IRGC 136064	CX236	2018
DANAU LAUT TAWAR::C1	IRGC 122029	IRIS_313-7668	2018
IR 2307-247-2-2-3::IRGC 77982-C1	IRGC 122092	IRIS_313-7689	2018
KOGONI 91-1::C1	IRGC 122140	IRIS_313-7719	2018
MADINIKA 1329::GERVEX 8366-C1	IRGC 124407	IRIS_313-7725	2018
SOM CAU 70 A::IRGC 8227-C1	IRGC 122254	IRIS_313-7778	2018
WAS 198-B-3-1-3::C1	IRGC 122287	IRIS_313-7815	2018
IRAT 112::C1	IRGC 122115	IRIS_313-7914	2018
ARIANA::GERVEX 396-C1	IRGC 121966	IRIS_313-8069	2018
CHANG LE SAN SHU ZAO::IRGC 63561-1	IRGC 126115	IRIS_313-8433	2018
UPRH 233::IRGC 61667-1	IRGC 125627	IRIS_313-8435	2018
ARC 18061::IRGC 47650-1	IRGC 127034	IRIS_313-8935	2018
FU ZAO XIAN::IRGC 63619-1	IRGC 125744	IRIS_313-8940	2018
XI GAN JING REN::IRGC 60035-1	IRGC 126014	IRIS_313-9184	2018
WP 65::IRGC 36526-1	IRGC 126013	IRIS_313-9227	2018
VARY MALADY MENA::IRGC 51555-1	IRGC 125931	IRIS_313-9388	2018
NCS 237::IRGC 62202-1	IRGC 125853	IRIS_313-9492	2018
RPW 9-4 (SS 1)::IRGC 50690-1	IRGC 126084	IRIS_313-9522	2018
ARC 11857::IRGC 40972-1	IRGC 125643	IRIS_313-9560	2018
ARC 10594::IRGC 12524-1	IRGC 125636	IRIS_313-9609	2018
I KUNG PAO::IRGC 114-1	IRGC 125770	IRIS_313-9758	2018
FUKUSHIMA MOCHI (GLUT)::IRGC 19296-1	IRGC 125743	IRIS_313-9884	2018
SUWEON 311::IRGC 61890-1	IRGC 125907	IRIS_313-10000	2018
GUI HUA ZAO::IRGC 68060-1	IRGC 125755	IRIS_313-10179	2018
DA NUO (ZHAN)::IRGC 72025-1	IRGC 125723	IRIS_313-10189	2018
TONG GU HONG::IRGC 81026-1	IRGC 125925	IRIS_313-10226	2018
ALTAMIRA 9::IRGC 116953-1	IRGC 127031	IRIS_313-10257	2018
INIAP 6::IRGC 117002-1	IRGC 127050	IRIS_313-10307	2018
HP 3319-2WX-6-4-1-B::IRGC 117331-1	IRGC 127049	IRIS_313-10353	2018
IR 80310-12-B-1-3-B::IRGC 117307-1	IRGC 125986	IRIS_313-10394	2018
YN 1353-3::IRGC 117363-1	IRGC 121160	IRIS_313-10423	2018
PACHCHAIPERUMAL 2462-11::IRGC 3474-1	IRGC 127698	IRIS_313-10476	2018

HSIEH DAU::IRGC 4727-1	IRGC 127419	IRIS_313-10504	2018
BELLO::IRGC 6658-2	IRGC 127992	IRIS_313-10542	2018
NAN TEO 14::IRGC 7304-1	IRGC 127645	IRIS_313-10561	2018
ALAGUSAMBA::IRGC 8944-2	IRGC 132315	IRIS_313-10610	2018
KHAO PHAE HOM::IRGC 23518-2	IRGC 132427	IRIS_313-10921	2018
BANDANG BUNGKUAKLAN::IRGC 24697-2	IRGC 127987	IRIS_313-10942	2018
HURANG ARISO LUTA::IRGC 26055-1	IRGC 127424	IRIS_313-10966	2018
AI NAN TSAO 39::IRGC 28461-2	IRGC 127924	IRIS_313-11038	2018
KWANG LU AI 4::IRGC 28480-2	IRGC 128093	IRIS_313-11039	2018
K 17-9-1-1::IRGC 36778-1	IRGC 127471	IRIS_313-11197	2018
CHITRAJ 14-134::IRGC 37837-2	IRGC 128015	IRIS_313-11220	2018
LIONG ORN::IRGC 38875-2	IRGC 128523	IRIS_313-11236	2018
BR 116-3B-53::IRGC 39559-2	IRGC 128001	IRIS_313-11241	2018
RP 1153-20-14::IRGC 40131-2	IRGC 132431	IRIS_313-11247	2018
IR 9560-2-6-3::IRGC 40451-1	IRGC 127441	IRIS_313-11250	2018
ARC 12757::IRGC 41095-2	IRGC 127944	IRIS_313-11257	2018
ARC 14737::IRGC 41729-2	IRGC 127961	IRIS_313-11271	2018
JASURE AUS::IRGC 43860-1	IRGC 127449	IRIS_313-11322	2018
LOBANG (WHITE)::IRGC 44548-2	IRGC 128097	IRIS_313-11338	2018
NAPDAI::IRGC 52009-1	IRGC 127647	IRIS_313-11446	2018
KALU T 139::IRGC 53670-1	IRGC 127482	IRIS_313-11489	2018
K 15591-4::IRGC 55043-1	IRGC 127470	IRIS_313-11506	2018
TV 30::IRGC 55221-1	IRGC 127870	IRIS_313-11507	2018
J 6 IR 520 (WC 693)::IRGC 57600-1	IRGC 127447	IRIS_313-11538	2018
NCS 477::IRGC 62299-1	IRGC 127659	IRIS_313-11641	2018
B 3913 B 16-20 ST 28::IRGC 63099-1	IRGC 127196	IRIS_313-11656	2018
JIN HUA 258::IRGC 63670-1	IRGC 127464	IRIS_313-11665	2018
RONG DAO 4::IRGC 63820-1	IRGC 127771	IRIS_313-11668	2018
SHANGYIPA::IRGC 64928-1	IRGC 128467	IRIS_313-11691	2018
DAW NOK KAEN::IRGC 65548-1	IRGC 127306	IRIS_313-11700	2018
JATI MANI::IRGC 66565-1	IRGC 127450	IRIS_313-11717	2018
QING ER XIAO 2::IRGC 67255-1	IRGC 127753	IRIS_313-11730	2018
SI CHAO 1::IRGC 67335-1	IRGC 127803	IRIS_313-11734	2018
MALAGKIT (PINELIPE)::IRGC 67444-1	IRGC 127595	IRIS_313-11736	2018
UGAGA::IRGC 67604-1	IRGC 127872	IRIS_313-11740	2018
GAO JIAO YING GAN ZHAN::IRGC 68053-1	IRGC 127378	IRIS_313-11748	2018
AUS 78-125::IRGC 69464-1	IRGC 132275	IRIS_313-11773	2018
E ZI 32::IRGC 70127-1	IRGC 128289	IRIS_313-11797	2018
E ZI 100::IRGC 70192-1	IRGC 127365	IRIS_313-11798	2018
BUENG MONG LENG WE::IRGC 71088-1	IRGC 128246	IRIS_313-11834	2018
BAI MI ZAI 7::IRGC 71940-1	IRGC 127202	IRIS_313-11852	2018
CHI GU::IRGC 71988-1	IRGC 127276	IRIS_313-11854	2018
HONG DU BAI::IRGC 72098-1	IRGC 127413	IRIS_313-11863	2018
LIU LI YOU::IRGC 72198-1	IRGC 127569	IRIS_313-11868	2018
YUN NAN ZHAN::IRGC 74272-1	IRGC 127908	IRIS_313-11911	2018
XIA HONG GU::IRGC 76803-1	IRGC 128504	IRIS_313-11954	2018
IR 19661-364-1-2-3::IRGC 78061-1	IRGC 127435	IRIS_313-11979	2018

IR 77298-14-1-2-10::G1	IRGC 126964	IRIS_313-15901	2018
IR 73571-3B-11-3-K2::G1	IRGC 126963	IRIS_313-15906	2018
AZUCENA	IRGC 135833	CX151	2019
Vandana	IRGC 136064	CX236	2019
Lijiangxintuanheigu	NA	CX282	2019
GAMBIAKA::GERVEX 7920-C1	IRGC 124392	IRIS_313-7620	2019
BOTOHAVANA 139::GERVEX 8237-C1	IRGC 124380	IRIS_313-7650	2019
BOTRA MAITSO::GERVEX 8389-C1	IRGC 121990	IRIS_313-7651	2019
CICA 8::C1	IRGC 122011	IRIS_313-7664	2019
IR 1561-228-3-3::IRGC 32627-C1	IRGC 122088	IRIS_313-7684	2019
IR 57924-24::IRTP 16675-C1	IRGC 122099	IRIS_313-7699	2019
ROJOKELY::GERVEX 8410-C1	IRGC 122220	IRIS_313-7758	2019
TSIPALA B 160::GERVEX 8378-C1	IRGC 124426	IRIS_313-7793	2019
VARY LAVA DE MAROVATO::GERVEX 839	IRGC 124428	IRIS_313-7799	2019
WAS 170-B-B-1-1::C1	IRGC 122284	IRIS_313-7807	2019
WAS 173-B-B-6-2-2::C1	IRGC 122285	IRIS_313-7808	2019
WAS 174-B-3-5::C1	IRGC 122286	IRIS_313-7809	2019
WAS 199-B-1-2-1::C1	IRGC 122288	IRIS_313-7816	2019
WAS 206-B-B-2-2-1::C1	IRGC 122290	IRIS_313-7820	2019
WAS 21-B-B-20-4-3-3::C1	IRGC 122291	IRIS_313-7824	2019
WAS 33-B-B-15-1-4-5::C1	IRGC 122292	IRIS_313-7826	2019
IR 72967-12-2-3::C1	IRGC 122111	IRIS_313-7911	2019
MEDUSA::GERVEX 323-C1	IRGC 122165	IRIS_313-8067	2019
ORIONE::GERVEX 333-C1	IRGC 122184	IRIS_313-8068	2019
CNA 4081::GERVEX 1494-C1	IRGC 122017	IRIS_313-8161	2019
A 201::GERVEX 1638-C1	IRGC 121959	IRIS_313-8173	2019
GIZA 178::GERVEX 1681-C1	IRGC 122060	IRIS_313-8212	2019
MERLE::GERVEX 1685-C1	IRGC 122168	IRIS_313-8215	2019
POKKALI::IRGC 8948-1	IRGC 126002	IRIS_313-8244	2019
AVO::IRGC 11000-1	IRGC 117430	IRIS_313-8288	2019
SURMATIYA::IRGC 74779-1	IRGC 126008	IRIS_313-8303	2019
JC 1::IRGC 9091-1	IRGC 117494	IRIS_313-8326	2019
ARC 15091::IRGC 43048-1	IRGC 126298	IRIS_313-8332	2019
PUILLIPINA KATARI::IRGC 77293-1	IRGC 126261	IRIS_313-8509	2019
MAKALIOKA STANDARD::IRGC 12768-1	IRGC 126240	IRIS_313-8595	2019
NEP HOA VANG::IRGC 40748-2	IRGC 117537	IRIS_313-8690	2019
DA 7::IRGC 5809-2	IRGC 124432	IRIS_313-8703	2019
HNANWA::IRGC 33118-1	IRGC 126209	IRIS_313-8751	2019
NIRGUNI::IRGC 61127-1	IRGC 126000	IRIS_313-8757	2019
E ZI 96::IRGC 70188-1	IRGC 125738	IRIS_313-8859	2019
PATALASAFED SUNGHAWADO::IRGC 6113	IRGC 125869	IRIS_313-8920	2019
BUCAYAB::IRGC 44357-1	IRGC 126974	IRIS_313-8948	2019
MAE MAI LUD NI::IRGC 66065-1	IRGC 125831	IRIS_313-9062	2019
BHOJON KOLPO::IRGC 31727-1	IRGC 125681	IRIS_313-9067	2019
LEUANG YAI 344::IRGC 651-1	IRGC 125826	IRIS_313-9112	2019
LEUANG 28-1-87::IRGC 874-1	IRGC 125822	IRIS_313-9114	2019
LEUANG YAI 2 B 72::IRGC 882-1	IRGC 126138	IRIS_313-9116	2019

KHAO LEUANG RAI::IRGC 48168-1	IRGC 125802	IRIS_313-9121	2019
KHAO MON::IRGC 60764-1	IRGC 125803	IRIS_313-9182	2019
GAM PAI 30-12-15::IRGC 831-1	IRGC 125747	IRIS_313-9302	2019
ARC 5840::IRGC 12144-1	IRGC 125656	IRIS_313-9424	2019
RP 9-4::IRGC 39735-1	IRGC 126004	IRIS_313-9566	2019
B 78-S81::IRGC 58415-1	IRGC 125662	IRIS_313-9574	2019
AI ZI HUNG::IRGC 51255-1	IRGC 126129	IRIS_313-9723	2019
NAN TE HAO::IRGC 59797-1	IRGC 125849	IRIS_313-9727	2019
BOHOTO BALOOCHESTAN::IRGC 66237-1	IRGC 125954	IRIS_313-10016	2019
EX MARABA GURUKU::IRGC 69582-1	IRGC 125737	IRIS_313-10047	2019
TAMASHIRO HIKARI::IRGC 74534-1	IRGC 125917	IRIS_313-10077	2019
FACAGRO 64::IRGC 82059-1	IRGC 125739	IRIS_313-10114	2019
PIN GAEW 56::IRGC 7887-1	IRGC 125871	IRIS_313-10134	2019
RD 15::IRGC 47705-1	IRGC 126003	IRIS_313-10151	2019
DENG DENG QI::IRGC 72036-1	IRGC 125726	IRIS_313-10190	2019
SICAN::IRGC 117029-1	IRGC 121119	IRIS_313-10285	2019
INIAP 10::IRGC 117000-1	IRGC 125969	IRIS_313-10314	2019
IR 52718-B-B-6-B-B-1-1::IRGC 117334-1	IRGC 126024	IRIS_313-10357	2019
IRGA 411-1-6-1F-A::IRGC 117344-1	IRGC 125990	IRIS_313-10402	2019
ARC 6052::IRGC 12196-1	IRGC 127163	IRIS_313-10664	2019
ARC 6218::IRGC 12255-1	IRGC 127164	IRIS_313-10666	2019
ARC 7236::IRGC 12335-1	IRGC 127168	IRIS_313-10667	2019
BARKHE TAULI::IRGC 16116-1	IRGC 127212	IRIS_313-10731	2019
ARC 10894::IRGC 21122-1	IRGC 127131	IRIS_313-10858	2019
ARC 14975::IRGC 41848-1	IRGC 127156	IRIS_313-11275	2019
ARC 15129::IRGC 41938-1	IRGC 127157	IRIS_313-11277	2019
ARC 13544::IRGC 42743-1	IRGC 127152	IRIS_313-11298	2019
AMARILLO::IRGC 47120-1	IRGC 127119	IRIS_313-11377	2019
BIR BAHADUR::IRGC 53889-1	IRGC 127230	IRIS_313-11491	2019
AR 133::IRGC 53942-1	IRGC 127126	IRIS_313-11493	2019
ADIALLO::IRGC 56264-1	IRGC 127106	IRIS_313-11523	2019
BHATA PYAGI::IRGC 60895-1	IRGC 127222	IRIS_313-11597	2019
ANADI WHITE::IRGC 61897-1	IRGC 127122	IRIS_313-11624	2019
AI JIAO AO FAN ZI::IRGC 67871-1	IRGC 127114	IRIS_313-11744	2019
AN FU ZHAN::IRGC 67878-1	IRGC 127123	IRIS_313-11745	2019
78 XUAN WU::IRGC 70475-1	IRGC 127101	IRIS_313-11806	2019
ASFALA::IRGC 70650-1	IRGC 127175	IRIS_313-11812	2019
AI DA::IRGC 72567-1	IRGC 127113	IRIS_313-11876	2019
AN NAN ZAO::IRGC 72576-1	IRGC 127125	IRIS_313-11877	2019
BAI RI XIAN::IRGC 72588-1	IRGC 127203	IRIS_313-11878	2019
FEDEARROZ 50::G1	IRGC 126957	IRIS_313-15896	2019
SANHUANGZHAN NO 2::G1	IRGC 126968	IRIS_313-15897	2019
IR 4630-22-2-5-1-3::G1	IRGC 126962	IRIS_313-15898	2019
IR 45427-2B-2-2B-1-1::G1	IRGC 126961	IRIS_313-15899	2019
IR 07F287::G1	IRGC 126960	IRIS_313-15900	2019
IRRI 146::G1	IRGC 126966	IRIS_313-15902	2019

Supplementary Information Table 4

Calculation of the WRI based on difference of shoot and root growth rate ratio (SRGR) of drought treatment and controls of all genotypes.

Genotype	SRGR (drought)	SRGR (control)	WRI	Ranking #
ADT 12	-1.022	4.467	-5.489	1
ARC 10754	-1.465	0.828	-2.293	6
ARC 18202	-0.967	0.148	-1.116	19
Ariana	-0.560	0.744	-1.304	15
Danau Laut Tawar	-0.600	0.400	-0.999	20
Daw Nok Kaen	-0.740	2.634	-3.374	3
Fallow Field Survivor 1	-1.110	0.373	-1.483	13
Fu Zao Xian	-4.559	0.351	-4.961	2
Gui Hua Zao	-0.860	0.709	-1.569	11
Holdi Gira	-0.665	0.556	-1.221	16
Hurang Ariso Luta	-0.503	1.625	-2.128	7
IR 2307-247-2-2-3	-1.582	0.136	-1.718	10
IR 64	-1.211	0.254	-1.465	14
Pai Chueh Chiu Liu	-2.000	0.733	-2.733	4
Perunel	-1.046	0.136	-1.182	17
PSBRC 68	-0.847	0.650	-1.497	12
Puttige	-0.736	1.206	-1.942	8
Race Perumal	-0.300	0.860	-1.160	18
Suweon 311	-2.482	0.189	-2.671	5
Xia Zhi Bai	-1.034	0.794	-1.828	9

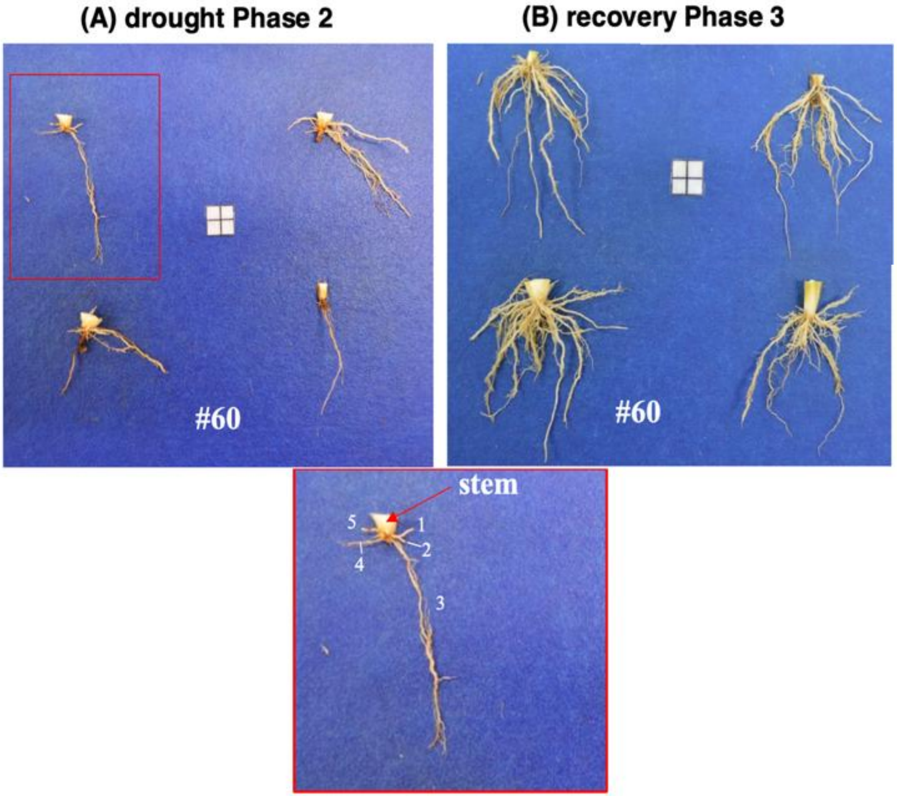

Supplementary Information Figure 1
Crown roots sampling example. Photos of 4 roots of the rice genotype “Suweon 311” plot #60. (A) before the re-watering (drought Phase 2 at M2) and (B) after re-watering (recovery Phase 3 at M3). Scale = 1 cm^2. (C) shows the crown counting of a single root as an example. Numbers indicate the axile roots counted (from nodes or seed) and arrows indicate seed, stem and lateral roots, which were not counted .

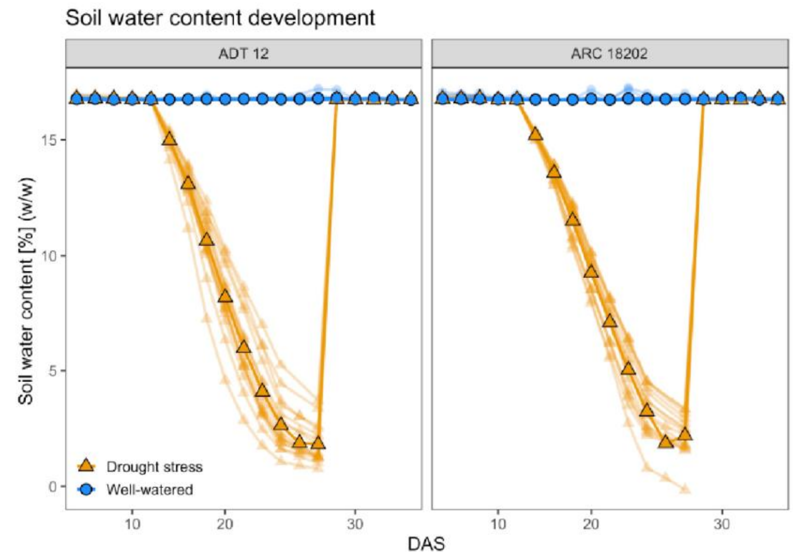

Supplementary Information Figure 2
Dynamic water content over the experimental timeframe. The soil-water content of the greenhouse experiment over time. Both lines are presented individually. The yellow triangles represent the drought stress measurement, the blue circles show the well-watered plants of each line. The thicker lines with black boarder represent the mean over all lines in the respective treatment, whereas the single plant measurements are shown in the background.

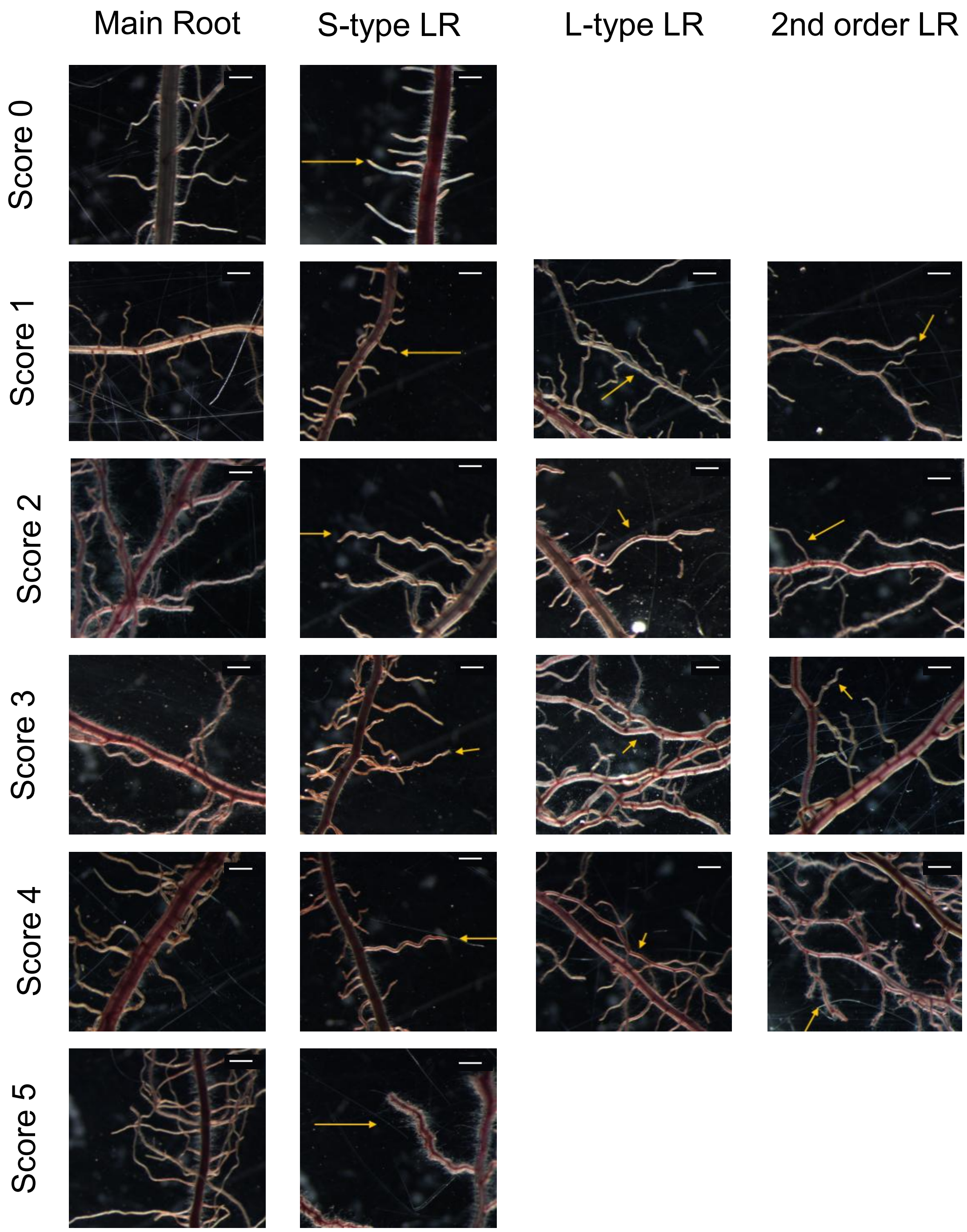

Supplementary Information Figure 3
Neutral Red Staining References. Length of scalebar in top right corner of the picture: 0.5 mm. The other root types were also scored via reference pictures, for the L-type lateral roots and 2nd order S-type lateral roots no scores of 0 and 5 were found.

a)

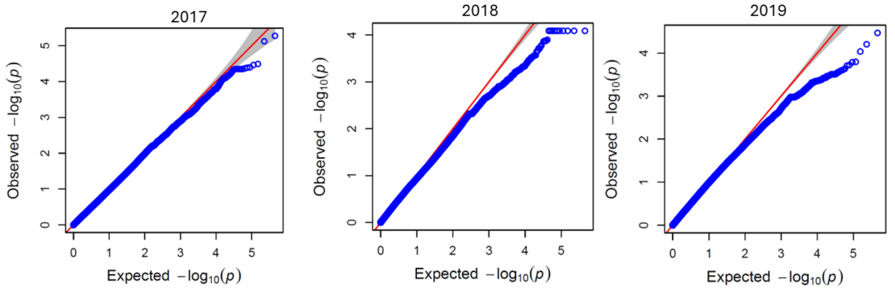

b)

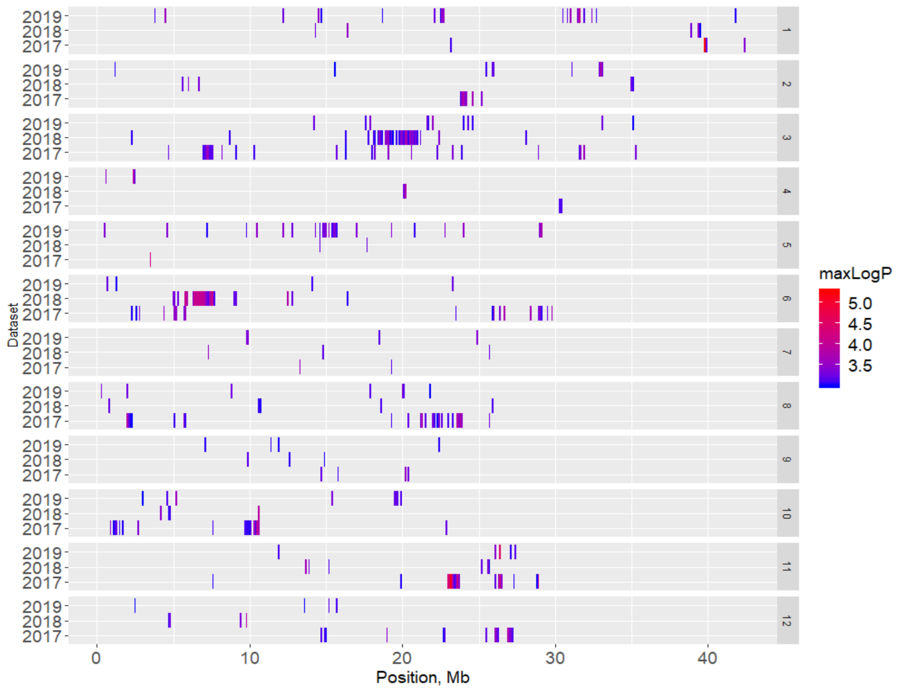

Supplementary Information Figure 4

GWAS peaks 2017-2019.

a) Q-Q plots from the RGR values in the 2017 (left), 2018 (middle), and 2019 (right) field experiments.

b) Colocation analysis of RGR (measured on two dates between the end of the drought stress treatment and about one week after rewatering) across three years of field screening.

Supplementary Information Table 5: Co-locating GWAS peaks and candidate genes between 2017 and 2018 analysis of RGR

Chr.	bin	2017	2018	start	end	Locus ID	Description/Function
3	***163***	***3.0254***	***3.052121***	***16300000***	***16399999***	***LOC_Os03g28330***	***sucrose synthase, putative, expressed***
						LOC_Os03g28350	expressed protein
						LOC_Os03g28360	expressed protein
						LOC_Os03g28380	retrotransposon protein, putative, unclassified, expressed
						LOC_Os03g28400	transport protein particle component, Bet3, domain containing protein, expressed
						LOC_Os03g28389	expressed protein
						LOC_Os03g28430	hypothetical protein
						LOC_Os03g28420	3-oxoacyl-synthase, putative, expressed
						LOC_Os03g28410	ribosomal protein S2, putative, expressed
						LOC_Os03g28910	expressed protein
3	182	3.254647	3.038123	18200000	18299999	LOC_Os03g31870	hypothetical protein
						LOC_Os03g31880	***SHORT-ROOT, putative, expressed***
						LOC_Os03g31910	transposon protein, putative, CACTA, En/Spm

							sub-class, expressed
						LOC_Os03g31934	hypothetical protein
						LOC_Os03g31944	expressed protein
						LOC_Os03g31950	keratin, putative, expressed
						LOC_Os03g31970	hypothetical protein
3	191	3.459711	3.518298	19100000	19199999	LOC_Os03g33460	retrotransposon protein, putative, unclassified, expressed
						LOC_Os03g33490	hypothetical protein
						LOC_Os03g33509	expressed protein
						LOC_Os03g33520	exo70 exocyst complex subunit, putative, expressed
						LOC_Os03g33550	hypothetical protein
						LOC_Os03g33570	calcineurin B-like protein 8, putative, expressed
						LOC_Os03g33580	mitotic checkpoint protein, putative, expressed
						LOC_Os03g33590	interferon-related developmental regulator, putative, expressed
3	206	3.400192	3.234773	20600000	20699999	LOC_Os03g37230	retrotransposon protein, putative, unclassified

						LOC_Os03g37240	retrotransposon protein, putative, unclassified, expressed
						LOC_Os03g37260	***pentatricopeptide, putative, expressed***
						LOC_Os03g37270	RNA recognition motif containing protein, putative, expressed
						LOC_Os03g37290	***cytochrome P450, putative, expressed***
						LOC_Os03g37320	expressed protein
						LOC_Os03g37330	KIN, antigenic determinant of recA protein, putative, expressed
						LOC_Os03g37340	expressed protein
6	51	3.506017	3.330215	5100000	5199999	LOC_Os06g10000	expressed protein
						LOC_Os06g10010	expressed protein
						LOC_Os06g10020	expressed protein
						LOC_Os06g10030	expressed protein
						LOC_Os06g10040	expressed protein
						LOC_Os06g10050	expressed protein
						LOC_Os06g10060	expressed protein
						LOC_Os06g10070	expressed protein
						LOC_Os06g10090	hypothetical protein
						LOC_Os06g10100	expressed protein
						LOC_Os06g10109	expressed protein
						LOC_Os06g10130	expressed protein

						LOC_Os06g10140	expressed protein
						LOC_Os06g10150	hypothetical protein
						LOC_Os06g10160	tyrosine protein kinase domain containing protein, putative, expressed
6	58	3.5762	4.090494	5800000	5899999	LOC_Os06g11060	expressed protein
						LOC_Os06g11070	expressed protein
						LOC_Os06g11080	hypothetical protein
						LOC_Os06g11090	CXE carboxylesterase, putative, expressed
						LOC_Os06g11120	expressed protein
						LOC_Os06g11130	gibberellin receptor GID1L2, putative, expressed
						LOC_Os06g11135	gibberellin receptor GID1L2, putative, expressed
						LOC_Os06g11140	pyridine nucleotide-disulphide oxidoreductase domain containing protein, expressed
						LOC_Os06g11150	DUF1645 domain containing protein, putative, expressed
						LOC_Os06g11160	expressed protein
						LOC_Os06g11170	formin-binding protein-related,

							putative, expressed
						LOC_Os06g11180	OsPOP12 - Putative Prolyl Oligopeptidase homologue, expressed
						LOC_Os06g11190	OsPOP13 - Putative Prolyl Oligopeptidase homologue, expressed
						LOC_Os06g11200	12-oxophytodienoate reductase, putative, expressed
						LOC_Os06g11210	12-oxophytodienoate reductase, putative, expressed
						LOC_Os06g11230	expressed protein
						LOC_Os06g11240	12-oxophytodienoate reductase, putative, expressed
10	106	3.730644	3.898613	10600000	10699999	LOC_Os10g20950	expressed protein
						LOC_Os10g20980	transposon protein, putative, CACTA, En/Spm sub-class, expressed
						LOC_Os10g20990	***ribosomal protein S12 containing protein, expressed***
						LOC_Os10g21000	nucleoside-triphosphatase,

							putative, expressed
						LOC_Os10g21020	expressed protein
						LOC_Os10g21040	expressed protein
						LOC_Os10g21050	cytochrome P450, putative, expressed
						LOC_Os10g21060	cytochrome P450, putative, expressed
						LOC_Os10g21090	ATP binding protein, putative, expressed

Supplementary Information Table 6: Co-locating GWAS peaks and candidate genes between 2017 and 2019 analysis of RGR.

Chr.	bin	2018	2019	start	end	Locus ID	Description/Function
8	20	3.81184	3.266792	2000000	2099999	LOC_Os08g04120	expressed protein
						LOC_Os08g04130	copine-6, putative, expressed
						LOC_Os08g04140	X8 domain containing protein, expressed
						LOC_Os08g04150	RNA polymerase II complex component SRB7, putative, expressed
						LOC_Os08g04160	expressed protein
						LOC_Os08g04170	zinc finger C-x8-C-x5-C-x3-H type

							family protein, expressed
						LOC_Os08g04180	tryptophan synthase beta chain 1, putative, expressed
						LOC_Os08g04190	homeobox and START domains containing protein, putative, expressed
						LOC_Os08g04200	expressed protein
						LOC_Os08g04210	cysteine-rich repeat secretory protein 55 precursor, putative, expressed
						LOC_Os08g04220	expressed protein
						LOC_Os08g04230	cysteine-rich repeat secretory protein 55 precursor, putative, expressed
						LOC_Os08g04240	cysteine-rich repeat secretory protein 55 precursor, putative, expressed
						LOC_Os08g04250	cysteine-rich repeat secretory protein 55 precursor, putative, expressed
						LOC_Os08g04260	expressed protein
						LOC_Os08g04270	WD domain, G-beta repeat domain containing protein, expressed
						LOC_Os08g04280	actin, putative, expressed
						LOC_Os08g04290	WD domain, G-beta repeat domain containing protein, expressed

11	261	3.171145	3.425608	26100000	26199999	LOC_Os11g43260	transposon protein, putative, CACTA, En/Spm sub-class, expressed
						LOC_Os11g43310	expressed protein
						LOC_Os11g43320	***NBS-LRR type disease resistance protein, putative, expressed***
						LOC_Os11g43340	expressed protein
						LOC_Os11g43360	tropinone reductase 2, putative, expressed
						LOC_Os11g43380	retrotransposon protein, putative, Ty3-gypsy subclass, expressed
						LOC_Os11g43390	expressed protein
11	264	4.082715	4.470322	26400000	26499999	LOC_Os11g43720	expressed protein
						LOC_Os11g43740	OsMADS68 - MADS-box family gene with MIKC* type-box, expressed
						LOC_Os11g43750	polygalacturonase, putative, expressed
						LOC_Os11g43760	***lipase class 3 family protein, putative, expressed***
						LOC_Os11g43770	Leucine Rich Repeat family protein, expressed
						LOC_Os11g43790	***DUF581 domain containing protein, expressed***
						LOC_Os11g43800	PPR repeat domain containing protein, putative, expressed
						LOC_Os11g43820	myristoyl-acyl carrier protein thioesterase,

							chloroplast precursor, putative, expressed
						LOC_Os11g43830	pectinesterase, putative, expressed
						LOC_Os11g43840	conserved hypothetical protein
						LOC_Os11g43860	sodium/calcium exchanger protein, putative, expressed

Supplementary Information Table 7: Co-locating GWAS peaks and candidate genes between 2018 and 2019 analysis of RGR

Chr.	bin	2018	2019	start	end	Locus ID	Description/Function
5	146	3.24874	3.08787	14600000	14699999	LOC_Os05g25194	metal cation transporter, putative, expressed
						LOC_Os05g25210	expressed protein
						LOC_Os05g25220	expressed protein
						LOC_Os05g25230	hypothetical protein
						LOC_Os05g25240	expressed protein
						LOC_Os05g25250	expressed protein
						LOC_Os05g25260	AP2 domain containing protein, expressed
						LOC_Os05g25270	expressed protein
						LOC_Os05g25280	retrotransposon protein, putative, unclassified, expressed

						LOC_Os05g25310	acyl-CoA synthetase protein, putative, expressed

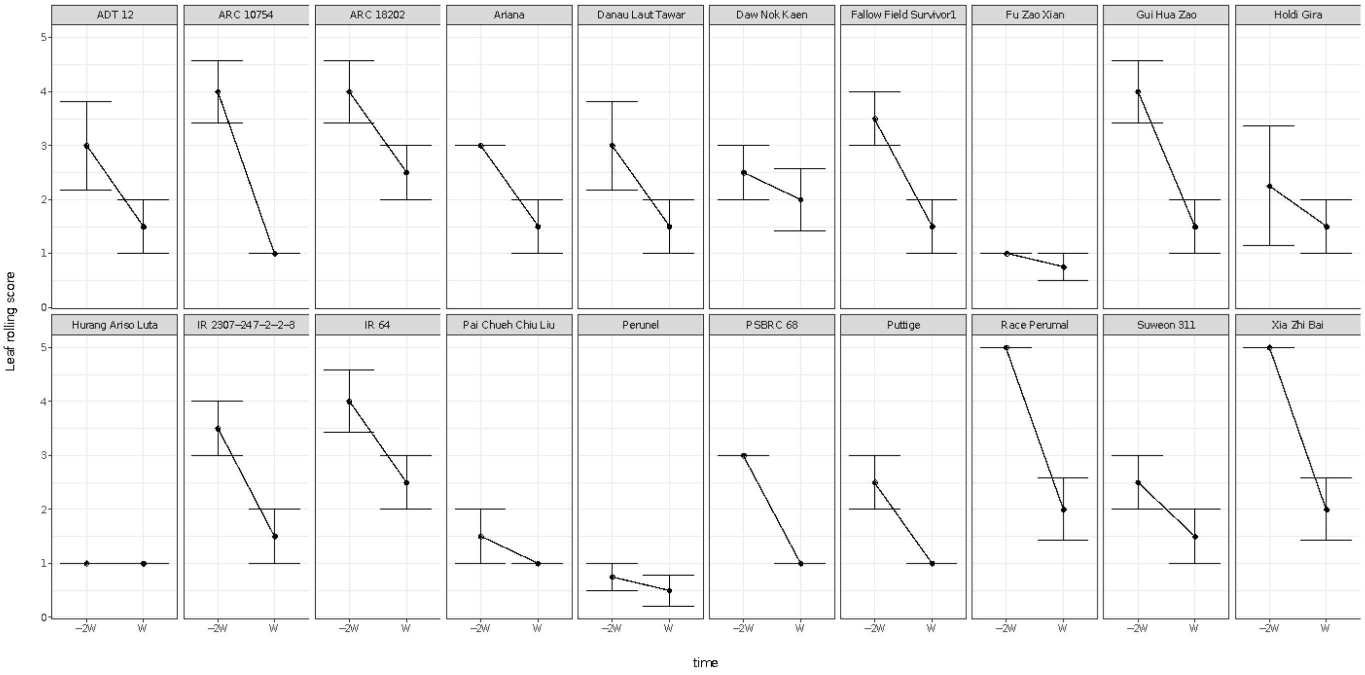

Supplementary Information Figure 5

Leaf rolling responses to drought per genotype. Scoring systems with numbers of leaf rolling (0-5) was given for each of the 20 genotypes individually at 33 DAS (before the re-watering drought - Phase 2) and at 36 DAS (after the re-watering on the same day in recovery - Phase 3).

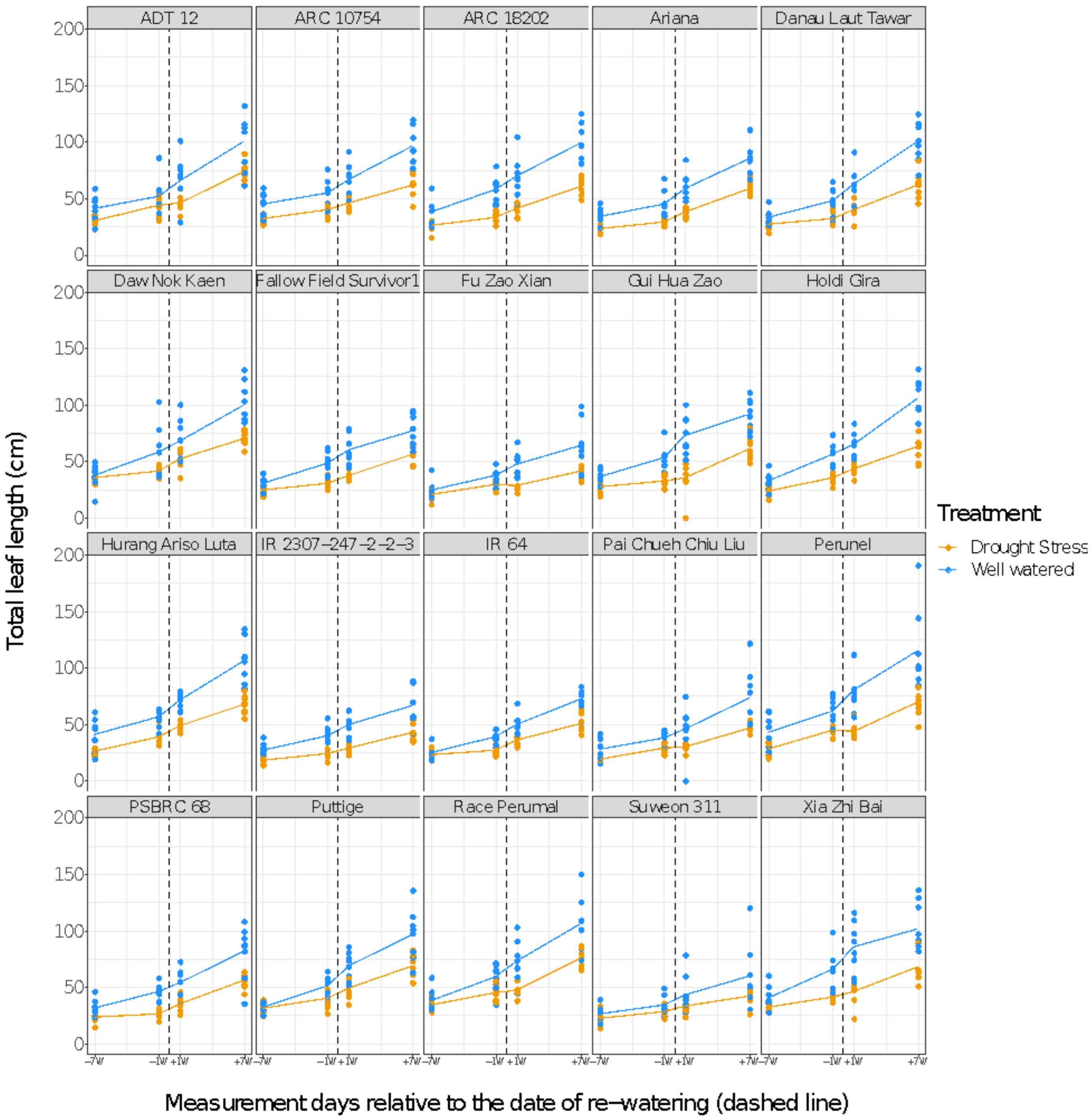

Supplementary Information Figure 6

Genotypic diversity in shoot responses to re-watering after drought. Non-invasively measured total leaf length on 4 measurement days of the 20 genotypes in two treatments (drought and control) in the course of the field experiment. Dots and light lines represent plants with n = 8, darker lines represent mean values. Time points (M1-M4) represent measurement days relative to the date of re-watering (dashed line). Genotypes were ordered alphabetically.

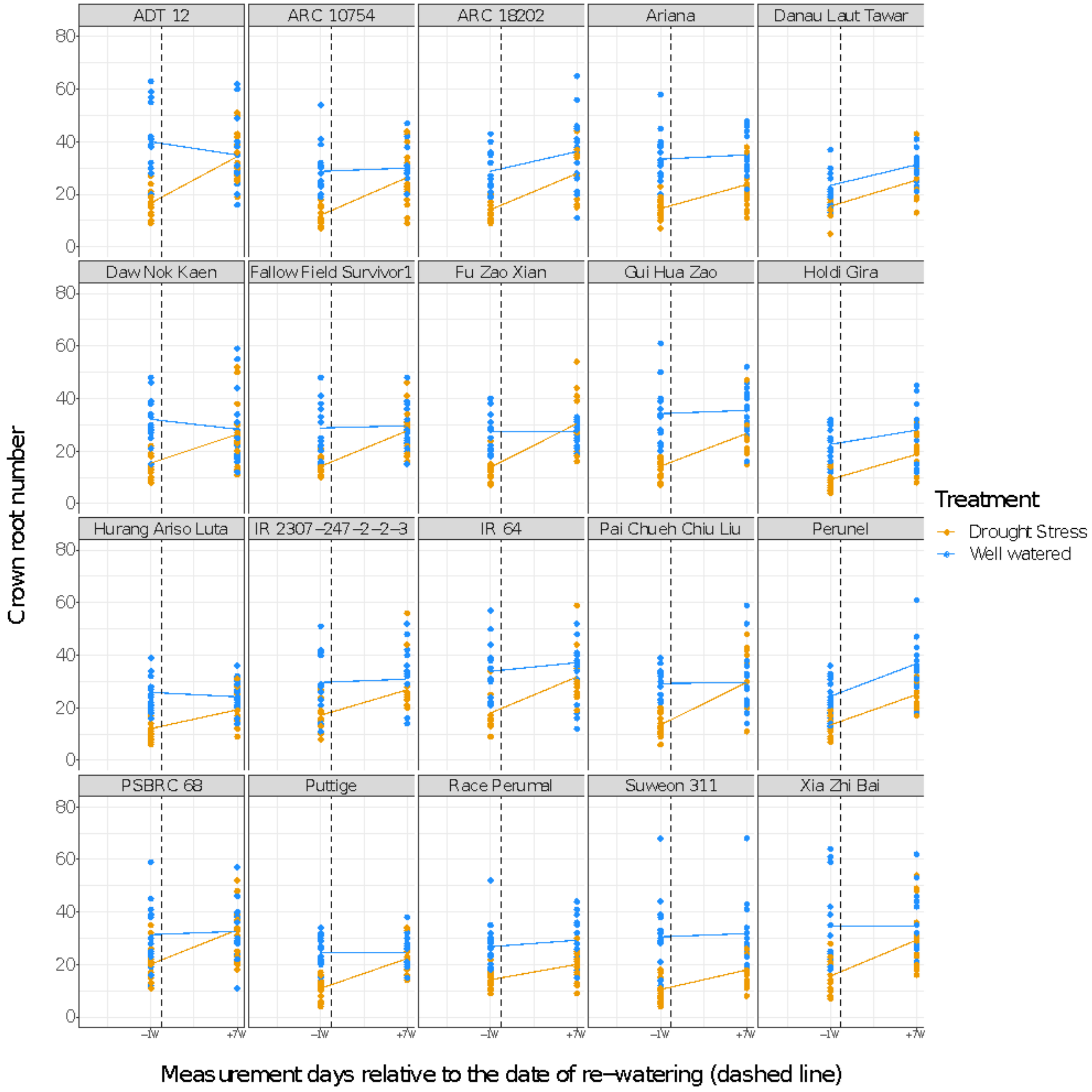

Supplementary Information Figure 7

Genotypic diversity in root responses to re-watering after drought. Destructively determined crown root number on 4 different time points of the 20 genotypes in two treatments (drought and control) over the course of the field experiment. Dots represent plants n = 16, lines represent mean value. Time points (M1-M4) represent measurement days in relative to the date of re-watering (dashed lines).

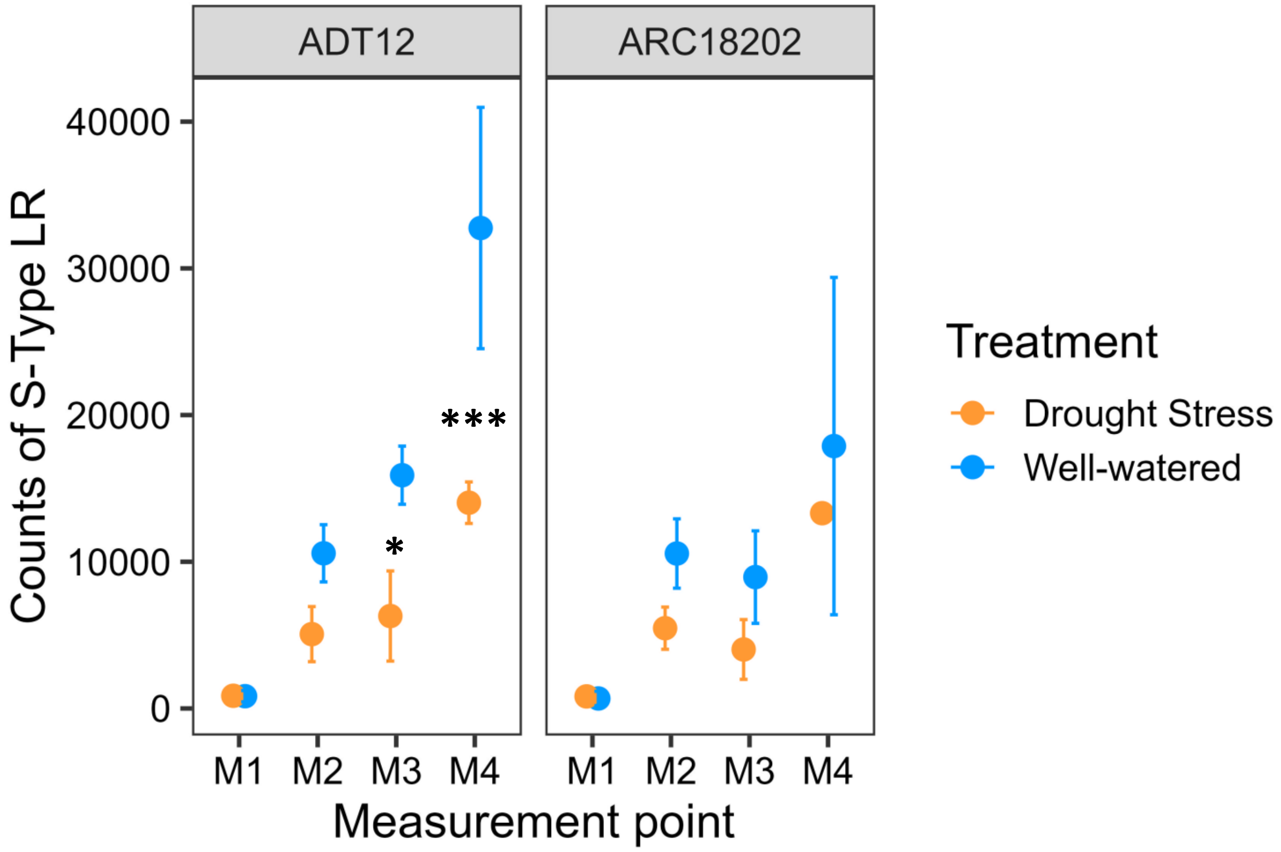

Supplementary Information Figure 8
Count of S-type lateral roots. Genotypic diversity in root responses to re-watering after drought. S-type LR counting based on WonRhizo Link analysis per Harvest. Colors indicate the treatment. M§ was removed based on small samplesize. Bars represent standart error of the mean (n=3). A Type-II-Anova, followed by a TukeyHSD test was used to test the differences within the genotypes (***=p<0.001, **=p<0.01,*=p<0.05).

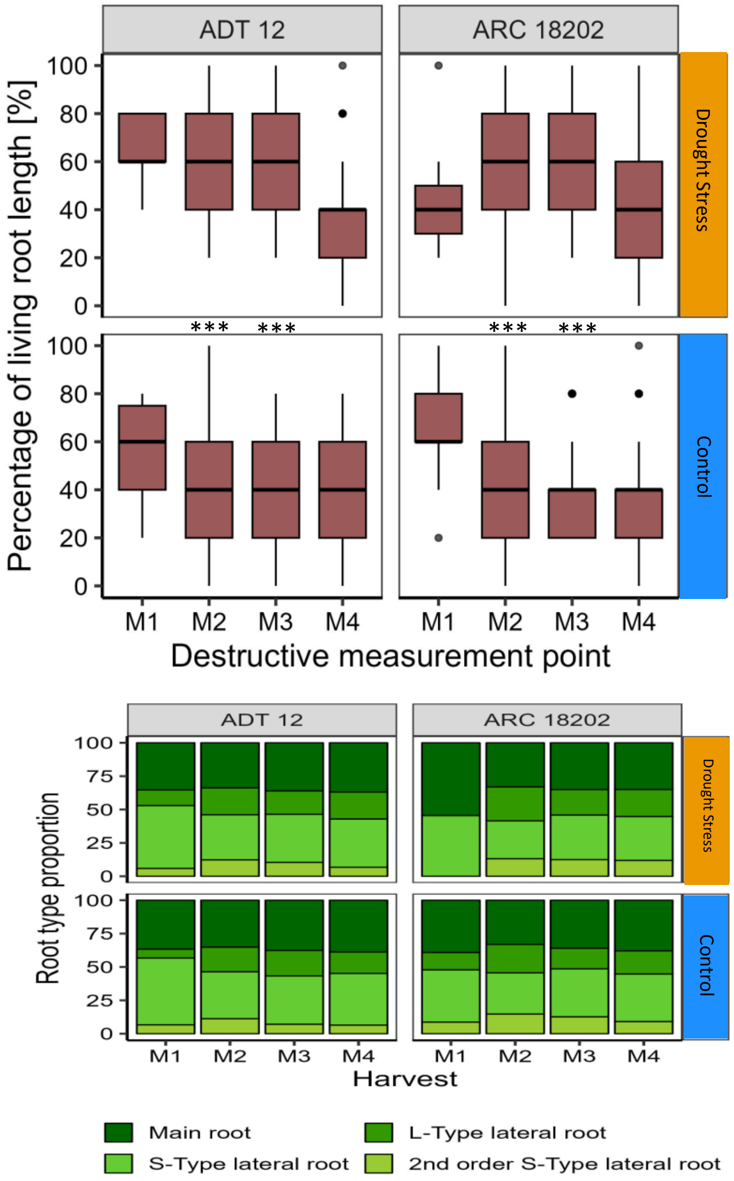

Supplementary Information Figure 9

Root staining.The living percentage of the total root length calculated out of the stainig score for every harvest splitted up by genotype and treatment. Comparisons within a genotype between different treatments were done with anova (Type2) and Tukey test and are, if significant, marked with stars. The samplesize ranges between 11 (M1,ARc18202,Drought) to 151(M2,ADT12,Well-watered). A Type-II-Anova, followed by a TukeyHSD test was used to test the differences within the genotypes (***=p<0.001, **=p<0.01,*=p<0.05).
